# Polygenic risk scores for prediction of breast cancer risk in Asian populations

**DOI:** 10.1016/j.gim.2021.11.008

**Published:** 2021-12-15

**Authors:** Weang-Kee Ho, Mei-Chee Tai, Joe Dennis, Xiang Shu, Jingmei Li, Peh Joo Ho, Iona Y. Millwood, Kuang Lin, Yon-Ho Jee, Su-Hyun Lee, Nasim Mavaddat, Manjeet K. Bolla, Qin Wang, Kyriaki Michailidou, Jirong Long, Eldarina Azfar Wijaya, Tiara Hassan, Kartini Rahmat, Veronique Kiak Mien Tan, Benita Kiat Tee Tan, Su Ming Tan, Ern Yu Tan, Swee Ho Lim, Yu-Tang Gao, Ying Zheng, Daehee Kang, Ji-Yeob Choi, Wonshik Han, Han-Byoel Lee, Michiki Kubo, Yukinori Okada, Shinichi Namba, The BioBank Japan Project, Sue K. Park, Sung-Won Kim, Chen-Yang Shen, Pei-Ei Wu, Boyoung Park, Kenneth R. Muir, Artitaya Lophatananon, Anna H. Wu, Chiu-Chen Tseng, Keitaro Matsuo, Hidemi Ito, Ava Kwong, Tsun L. Chan, Esther M. John, Allison W. Kurian, Motoki Iwasaki, Taiki Yamaji, Sun-Seog Kweon, Kristan J. Aronson, Rachel A. Murphy, Woon-Puay Koh, Chiea-Chuen Khor, Jian-Min Yuan, Rajkumar Dorajoo, Robin G. Walters, Zhengming Chen, Liming Li, Jun Lv, Keum-Ji Jung, Peter Kraft, Paul D.B. Pharoah, Alison M. Dunning, Jacques Simard, Xiao-Ou Shu, Cheng-Har Yip, Nur Aishah Mohd Taib, Antonis C. Antoniou, Wei Zheng, Mikael Hartman, Douglas F. Easton, Soo-Hwang Teo

**Affiliations:** 1 School of Mathematical Sciences, Faculty of Science and Engineering, University of Nottingham Malaysia, Selangor, Malaysia; 2 Cancer Research Malaysia, Selangor, Malaysia; 3 Centre for Cancer Genetic Epidemiology, Department of Public Health and Primary Care, University of Cambridge, Cambridge, United Kingdom; 4 Division of Epidemiology, Department of Medicine, Vanderbilt Epidemiology Center, Vanderbilt-Ingram Cancer Center, Vanderbilt University, Nashville, TN; 5 Department of Epidemiology & Biostatistics, Memorial Sloan Kettering Cancer Center, New York, NY; 6 Department of Surgery, University Surgical Cluster, National University Hospital, Singapore, Singapore; 7 Genome Institute of Singapore, Laboratory of Women’s Health and Genetics, Singapore, Singapore; 8 Nuffield Department of Population Health, University of Oxford, Oxford, United Kingdom; 9 MRC Population Health Research Unit, University of Oxford, Oxford, United Kingdom; 10 Department of Epidemiology, Harvard T.H. Chan School of Public Health, Boston, MA; 11 Graduate School of Public Health, Yonsei University, Seoul, Korea; 12 Biostatistics Unit, The Cyprus Institute of Neurology & Genetics, Ayios Dometios, Cyprus; 13 Cyprus School of Molecular Medicine, The Cyprus Institute of Neurology & Genetics, Ayios Dometios, Cyprus; 14 Biomedical Imaging Department, Faculty of Medicine, University of Malaya, Kuala Lumpur, Malaysia; 15 Department of Breast Surgery, Singapore General Hospital, Singapore, Singapore; 16 Division of Surgery and Surgical Oncology, National Cancer Center Singapore, Singapore, Singapore; 17 Department of General Surgery, Sengkang General Hospital, Singapore, Singapore; 18 Division of Breast Surgery, Changi General Hospital, Singapore, Singapore; 19 Department of General Surgery, Tan Tock Seng Hospital, Singapore, Singapore; 20 KK Breast Department, KK Women’s and Children’s Hospital, Singapore, Singapore; 21 State Key Laboratory of Oncogene and Related Genes & Department of Epidemiology, Shanghai Cancer Institute, Renji Hospital, Shanghai Jiaotong University School of Medicine, Shanghai, China; 22 Shanghai Municipal Center for Disease Control and Prevention, Shanghai, China; 23 Department of Preventive Medicine, Seoul National University College of Medicine, Seoul, Korea; 24 Cancer Research Institute, Seoul National University, Seoul, Korea; 25 Department of Biomedical Sciences, Seoul National University Graduate School, Seoul, Korea; 26 Institute of Health Policy and Management, Medical Research Center, Seoul National University, Seoul, Korea; 27 Department of Surgery, Seoul National University College of Medicine, Seoul, South Korea; 28 RIKEN Center for Integrative Medical Sciences (IMS), Yokohama, Japan; 29 Department of Statistical Genetics, Graduate School of Medicine, Faculty of Medicine, Osaka University, Suita, Japan; 30 Integrated Frontier Research for Medical Science Division, Institute for Open and Transdisciplinary Research Initiatives, Osaka University, Suita, Japan; 31 Laboratory of Statistical Immunology, Immunology Frontier Research Center (WPI-IFReC), Osaka University, Suita, Japan; 32 Institute of Medical Science, The University of Tokyo, Tokyo, Japan; 33 Integrated Major in Innovative Medical Science, Seoul National University College of Medicine, Seoul, Korea; 34 Department of Surgery, Daerim Saint Mary's Hospital, Seoul, Korea; 35 Institute of Biomedical Sciences, Academia Sinica, Taipei, Taiwan; 36 Department of Medicine, Hanyang University College of Medicine, Seoul, Korea; 37 Division of Population Health, Health Services Research and Primary Care, School of Health Sciences, The University of Manchester, Manchester, United Kingdom; 38 Department of Preventive Medicine, Keck School of Medicine, University of Southern California, Los Angeles, CA; 39 Division of Cancer Epidemiology and Prevention, Aichi Cancer Center Research Institute, Nagoya, Japan; 40 Division of Cancer Epidemiology, Nagoya University Graduate School of Medicine, Nagoya, Japan; 41 Division of Cancer Information and Control, Aichi Cancer Center Research Institute, Nagoya, Japan; 42 Division of Descriptive Cancer Epidemiology, Nagoya University Graduate School of Medicine, Nagoya University, Nagoya, Japan; 43 Hong Kong Hereditary Breast Cancer Family Registry, Cancer Genetics Centre, Happy Valley, Hong Kong; 44 Department of Surgery, The University of Hong Kong, Pok Fu Lam, Hong Kong; 45 Department of Surgery, Hong Kong Sanatorium & Hospital, Happy Valley, Hong Kong; 46 Department of Pathology, Hong Kong Sanatorium & Hospital, Happy Valley, Hong Kong; 47 Division of Oncology, Department of Medicine, Stanford Cancer Institute, Stanford University School of Medicine, Stanford, CA; 48 Department of Epidemiology & Population Health, Stanford University School of Medicine, Stanford, CA; 49 Division of Epidemiology, Center for Public Health Sciences, National Cancer Center, Tokyo, Japan; 50 Department of Preventive Medicine, Chonnam National University Medical School, Hwasun, Korea; 51 Jeonnam Regional Cancer Center, Chonnam National University Hwasun Hospital, Hwasun, Korea; 52 Department of Public Health Sciences, and Cancer Research Institute, School of Medicine, Queen’s University, Kingston, Ontario, Canada; 53 Cancer Control Research, BC Cancer, Vancouver, British Columbia, Canada; 54 School of Population and Public Health, Faculty of Medicine, The University of British Columbia, Vancouver, British Columbia, Canada; 55 Healthy Longevity Translational Research Programme, Yong Loo Lin School of Medicine, National University of Singapore, Singapore, Singapore; 56 Singapore Institute for Clinical Sciences, Agency for Science, Technology and Research (A*STAR), Singapore, Singapore; 57 Genome Institute of Singapore, Agency for Science, Technology and Research (A*STAR), Singapore, Singapore; 58 Division of Cancer Control and Population Sciences, UPMC Hillman Cancer Center, University of Pittsburgh, Pittsburgh, PA; 59 Department of Epidemiology, Graduate School of Public Health, University of Pittsburgh, Pittsburgh, PA; 60 Health Services and Systems Research, Duke-NUS Medical School, Singapore, Singapore; 61 Department of Epidemiology and Biostatistics, School of Public Health, Peking University Health Science Center, Beijing, China; 62 Institute for Health Promotion, Graduate School of Public Health, Yonsei University, Seoul, Korea; 63 Department of Biostatistics, Harvard T.H. Chan School of Public Health, Boston, MA; 64 Centre for Cancer Genetic Epidemiology, Department of Oncology, University of Cambridge, Cambridge, United Kingdom; 65 Genomics Center, CHU de Québec-UniversitéLaval Research Center, Quebec City, Quebec, Canada; 66 Subang Jaya Medical Centre, Selangor, Malaysia; 67 Department of Surgery, Faculty of Medicine, University of Malaya Centre, UM Cancer Research Institute, Kuala Lumpur, Malaysia; 68 Department of Surgery, Yong Loo Lin School of Medicine, National University of Singapore and National University Health System, Singapore, Singapore; 69 Saw Swee Hock School of Public Health, National University of Singapore and National University Health System, Singapore, Singapore

**Keywords:** Breast cancer, Genetic, Polygenic risk score, Risk prediction

## Abstract

**Purpose:**

Non-European populations are under-represented in genetics studies, hindering clinical implementation of breast cancer polygenic risk scores (PRSs). We aimed to develop PRSs using the largest available studies of Asian ancestry and to assess the transferability of PRS across ethnic subgroups.

**Methods:**

The development data set comprised 138,309 women from 17 case-control studies. PRSs were generated using a clumping and thresholding method, lasso penalized regression, an Empirical Bayes approach, a Bayesian polygenic prediction approach, or linear combinations of multiple PRSs. These PRSs were evaluated in 89,898 women from 3 prospective studies (1592 incident cases).

**Results:**

The best performing PRS (genome-wide set of single-nucleotide variations [formerly single-nucleotide polymorphism]) had a hazard ratio per unit SD of 1.62 (95% CI = 1.46-1.80) and an area under the receiver operating curve of 0.635 (95% CI = 0.622-0.649). Combined Asian and European PRSs (333 single-nucleotide variations) had a hazard ratio per SD of 1.53 (95% CI = 1.37-1.71) and an area under the receiver operating curve of 0.621 (95% CI = 0.608-0.635). The distribution of the latter PRS was different across ethnic subgroups, confirming the importance of population-specific calibration for valid estimation of breast cancer risk.

**Conclusion:**

PRSs developed in this study, from association data from multiple ancestries, can enhance risk stratification for women of Asian ancestry.

## Introduction

Genetic inheritance is an important risk factor for breast cancer.^1^ Rare pathogenic variants in several susceptibility genes, including *BRCA1, BRCA2* and *PALB2*, confer increased risks of breast cancer^2^; however, a majority of the genetic variations in risk is polygenic owing to the fact that a large number of genetic variants combine, in which each genetic variant confers a small increase in risk. The effects of these variants can be summarized as polygenic risk scores (PRSs).^3,4^ Mavaddat et al^3^ developed and validated a 313 variant breast cancer PRS (PRS-313), using data from European-ancestry women in the Breast Cancer Association Consortium (BCAC).^4,5^ The lifetime risk of breast cancer was estimated to be 2.6% for women in the lowest 1% of the PRS-313 distribution and approximately 32% for women in the highest 1%; the latter group would be classified as at high-risk of developing breast cancer according to the National Institute for Health and Care Excellence and other clinical management guidelines.^3^ This shows the potential of PRS to improve quantification of risk and consequently optimize breast cancer screening and prevention strategies.^6^

Non-European populations are under-represented in genetic studies, and this could limit PRS adoption and applicability^7–9^ and exacerbate health disparities.^10^ This is important for ethnic minorities in high income countries, where clinical evaluation of the European PRS-313 is already underway but perhaps more so in low- and middleincome countries, where there is an urgent need to develop breast cancer screening strategies to address rapidly rising breast cancer incidence and high breast cancer mortality.^11^

Asians constitute more than half of the world’s population and are facing a dramatic increase in breast cancer incidence^12,13^ but make up only 15% of participants in the breast cancer genome-wide association studies (GWAS). Efforts to develop breast cancer PRS specifically for Asian populations have so far been limited. In our previous work, we showed that PRS-313, developed for Europeans, was predictive of breast cancer risk in Asian populations, although the effect size was somewhat smaller than that reported in European populations.^14^ However, an important outstanding question is whether a more predictive PRS using Asian data can be developed. Thus far, the largest study to attempt this involved 23,372 women of Asian ancestry. This study evaluated previously published breast cancer risk single-nucleotide variations (SNVs) (formerly singlenucleotide polymorphisms) and took forward SNVs that were significantly associated with breast cancer risk in Asians (*P* < .05) for PRS derivation, resulting in a 44-SNV PRS.^15^ Although predictive, we have shown in our previous work that the discriminatory power of 44-SNV PRS (area under the receiver operating curve [AUC] = 0.586) was much lower than PRS-313 (AUC = 0.617), derived from European ancestry women, for predicting breast cancer risk in Asian women.^14^

In this study, our objectives were twofold: (1) to develop improved breast cancer PRSs using data from Asian populations and to validate their performance in prospective cohorts using the largest available breast cancer genetic study of Asian ancestry and (2) to assess the transferability of PRSs across Asian ethnic subgroups.

## Materials and Methods

### Study populations

The study population was divided into training, validation, and testing data sets. The training data sets included (1) set 1, which comprised 22,013 invasive cases and 22,114 controls of East Asian ancestry from studies participating in BCAC and Asia Breast Cancer Consortium (where GWAS summary statistics of SNVs significant up to *P* < .0001 were available); (2) set 2, which comprised 16,680 invasive cases and 83,414 controls of East Asian ancestry from studies participating in BCAC together with BioBank Japan (where GWAS summary statistics were available); and (3) set 3, which comprised 122,977 invasive cases and 105,974 controls of European ancestry participating in BCAC^4^(where GWAS summary statistics were available). The validation data set comprised (1) 6392 invasive cases and 6638 controls of Chinese or Malay ancestry and (2) 585 invasive cases and 1018 controls of Indian ancestry participating in 2 multiethnic case-control studies: the Malaysian Breast Cancer Genetics study and the Singapore Breast Cancer Cohort study. The testing data set comprised 89,898 women (1595 incident cases) from 3 prospective cohorts of East Asian ancestry: the Singapore Chinese Health Study,^16^ the China Kadoorie Biobank,^17^ and the Korean Cancer Prevention Study Biobank.^18^ Supplemental Table 1 summarizes the study design, genotyping arrays, and the sample size in each study. Genotype calling, quality control procedures, and imputation methods have been described previously.^4,19–23^ Ancestry informative principal components (PCs) were available for Asian ancestry samples in the BCAC and validation data sets generated using methods as previously described.^24^ See Supplemental Methods for more details.

All studies were approved by the relevant institutional ethics committees and review boards, and all participants provided written informed consent.

### Statistical methods

PRSs were given by the following equation:

$$\text{PRS}=\beta_{1}{\text{x}}_{1}+\beta_{2}{\text{x}}_{2}+\ldots +\beta_{\text{k}}{\text{x}}_{\text{k}}+\ldots +\beta_{\text{m}}{\text{x}}_{\text{m}}$$

where x_k_ is the allele dosage for SNV k, β_k_ is the corresponding weight, and m is the total number of SNVs. PRSs were standardized to have unit SD in the control subjects. Logistic regression models, adjusted for the first 10 PCs and study, were used to estimate odds ratios (ORs) for association between the standardized PRSs and breast cancer risk in the validation set. The studies in the validation set were genotyped in 2 batches and hence treated as different strata for the purposes of adjustment. Cox proportional hazard model, adjusted for the first 2 PCs for Singapore Chinese Health Study (SCHS) and Korean Cancer Prevention Study-II and the first 12 PCs for China Kadoorie Biobank, was used to estimate hazard ratios per SD (HR_perSD_) for the association between the PRS and breast cancer risk in the test set. The discrimination of PRS was assessed using AUC. The HR_perSD_ and AUC were obtained individually for each study and combined using a fixed-effect meta-analysis. Test of heterogeneity between studies were obtained using *rma*() command in the *metafor* package in R version 3.6.1.^25^

**Figure 1:**
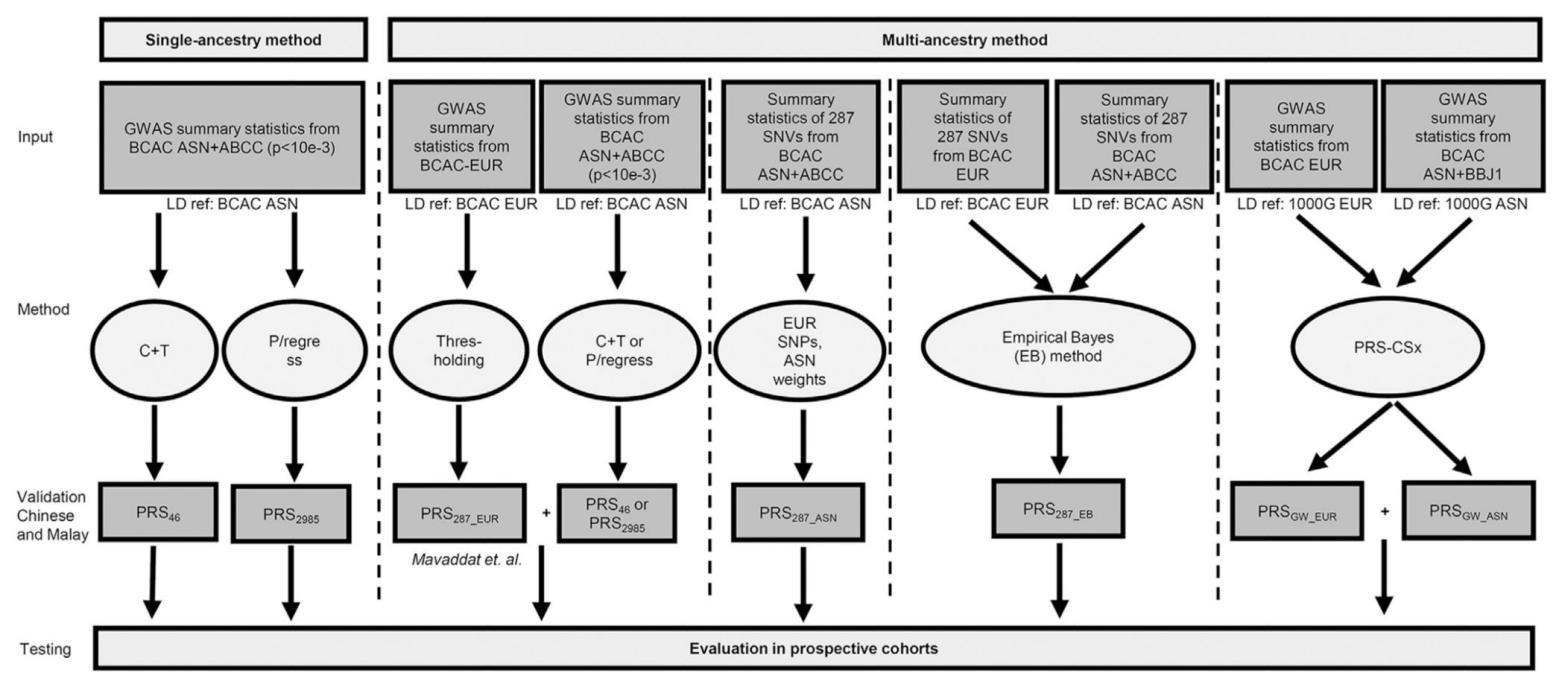
Overview of methods for PRSs development. Inputs are summary statistics from the meta-analysis of multiple GWAS data sets—BCAC ASN + ABCC denotes training data set 1, BCAC ASN + BBJ denotes training data set 2, and BCAC-EUR denotes training data set 3 as described in the method section. LD ref: BCAC ASN denotes OncoArray studies in which BCAC Asian studies were used as reference panel; LD ref: BCAC EUR denotes BCAC studies in which European ancestries were used as reference panel; 1000G ASN and 1000G EUR denote the Asian and European samples, respectively, in 1000 Genomes Project. Figure 1 shows methods using East Asian–ancestry women (Chinese and Malays), and as an example, same methods were applied to South Asian–ancestry women in the validation data set. ABCC, Asia Breast Cancer Consortium; ASN, Asian; BBJ, The BioBank Japan Project; BCAC, Breast Cancer Association Consortium; C + T, clumping and thresholding; EUR, European; GWAS, genome-wide association study; LD ref, reference panel for linkage disequilibrium; PRS, polygenic risk score; SNV, single-nucleotide variation.

The approaches for SNVs selection to be included in PRS and the corresponding weights are described in subsequent sections. Figure 1 and Supplemental Figure 1 summarizes the methods and data set. The lists of SNVs and the weights for the PRS computation are given in Supplemental Tables 2 to 4.

### Clumping and thresholding approach

Training data set 1 was used in these analyses. SNVs clumping (within 1 megabase pair windows) was conducted to remove highly correlated SNVs (pairwise correlation *r* ^2^ > 0.9); the SNV with the lowest *P* value for association in the correlated pairs was retained, resulting in 3050 SNVs. SNVs were further clumped within prespecified clumping window sizes and threshold of a correlation *r* ^2^. PRSs were then computed using the subset of SNVs that were significant at a prespecified *P* value threshold (set at 5 × 10^−8^ and then increased in steps of 10^−10^ up to 10^−3^). The PRS with the highest AUC in the validation data set was selected as the best PRS. The clumping and derivation of PRSs were performed using PRSice v2.11,^26^ whereas the AUCs for PRSs were generated using the pROC package in R version 3.6.1.

To account for the joint effect of SNVs used to derive the best PRS, we computed the optimal weight, from the summary statistics, for SNV j using the following formula:

(1) $$\gamma_{j}=\gamma_{j}^{\prime }/\sqrt{2p_{j}(1-p_{j})}$$

where *γ*′ = *R* ^−1^ *β′* , *R* is the correlation matrix between the SNV genotypes, *β′* is the predicted normalized marginal effect sizes of the SNVs, and *p_j_* is the effect allele frequency of SNV *j* (see Supplemental Methods).

### Lasso penalized regression

All 3050 SNVs described in clumping and thresholding (C + T) section were included in these analyses, together with genotype data from Asian controls in BCAC OncoArray studies for calculating linkage disequilibrium among SNVs. The analyses were run using the package *lassosum* in R^27^ across different values of the penalty and shrinkage parameters, and the PRS giving the highest correlation between PRS and the disease status (default metric in the method) in the validation data set was selected.

### Linear combination of European PRS with Asian PRS

Of the 313 SNVs included in PRS developed for European women,^3^ only 287 SNVs with imputation info score > 0.9 in validation data set were retained for subsequent analyses. Reported weights^3^ were used to derive the European PRS (hereafter denoted as PRS_287_EUR_). Asian PRSs generated from C + T or lasso penalized regression were linearly combined with PRS_287_EUR._ The relative contribution of each PRS were estimated by logistic regression using the validation data set.

### Reweighting of European-based PRS

We considered 2 sets of weights for PRS derivation using the 287 SNVs: (1) Asian weights estimated from the training data set 1, taking into account the correlation between SNVs using equation (1) (hereafter denoted as PRS_287_ASN_), and (2) weights based on a combination of the Asian and European weights using an Empirical Bayes (EB) approach (hereafter denoted as PRS_287_EB_), where the optimal weight is given by the following equation:

$$\beta_{j,EB}=\beta_{jA,\;EB}/\sqrt{2p_{j}(1-p_{j})}.$$

Here, *β_jA, EB_* is the estimated posterior effect sizes in Asians given the data and *p_j_* is the allele frequency for SNV *j* (see Supplemental Methods). Other approaches to combine European- and Asian-specific weights were also explored, including fixed effect meta-analysis, but only the method that gave the best AUC is presented in this study.

We also considered linear combinations of the reweighted European PRSs with Asian PRSs generated from C + T method or lasso penalized regression (as described before).

### Bayesian polygenic prediction approach (PRS-CSx)

Training sets 2 and 3 were used as training data sets for PRS-CSx^28^ together with Asians and Europeans in the 1000 Genomes Phase 3 project as linkage disequilibrium reference panels.^29^ PRSs generated using European- (hereafter denoted as PRS_GW_EUR_) and Asian-specific posterior weights (hereafter denoted as PRS_GW_ASN_) were linearly combined (hereafter denoted as PRS_GW_EUR_ + PRS_GW_ASN_) in the validation data set. The analyses were repeated across a range of global shrinkage parameter (*φ*), and the *φ* that gave the linear combination of PRSs with the highest AUC in the validation data set was selected as the optimal *φ*. Analyses were run using the published Python code-based tool in Github.^27^

### PRSs for the South Asian population

The predictive performance of PRSs developed for East Asian–ancestry women in Indian-ancestry women were assessed using AUC and OR per SD (OR_perSD_). Given the much smaller sample size of Indian-ancestry women, we did not attempt to generate a South Asian–specific PRS, but we considered estimating the weights in the linear combinations of multiple PRSs using the South Asian validation data set.

### Absolute risk of breast cancer by PRS percentiles

The age-specific absolute risks of developing breast cancer in each PRS percentile were obtained by constraining to the incidence of overall population breast cancer incidence (see Supplemental Methods). The details of these methods have been described previously.^3^ We calculated lifetime and 10-year absolute risks using Singaporean mortality and breast cancer incidence in 2017.^30,31^ For birth-cohort specific incidences, age-specific breast cancer incidences for the 1960-1969 and 1970-1979 birth cohorts were calculated using the data on breast cancer incidence in Singapore from 1968 to 2017.^30^ For women born between 1980 and 1989, incidences could only be calculated up to age 35, and hence, breast cancer incidences were projected by assuming an annual increase in breast cancer incidence of 3.9%.^32^

## Results

### Genetic diversity within Asian populations

**Figure 2:**
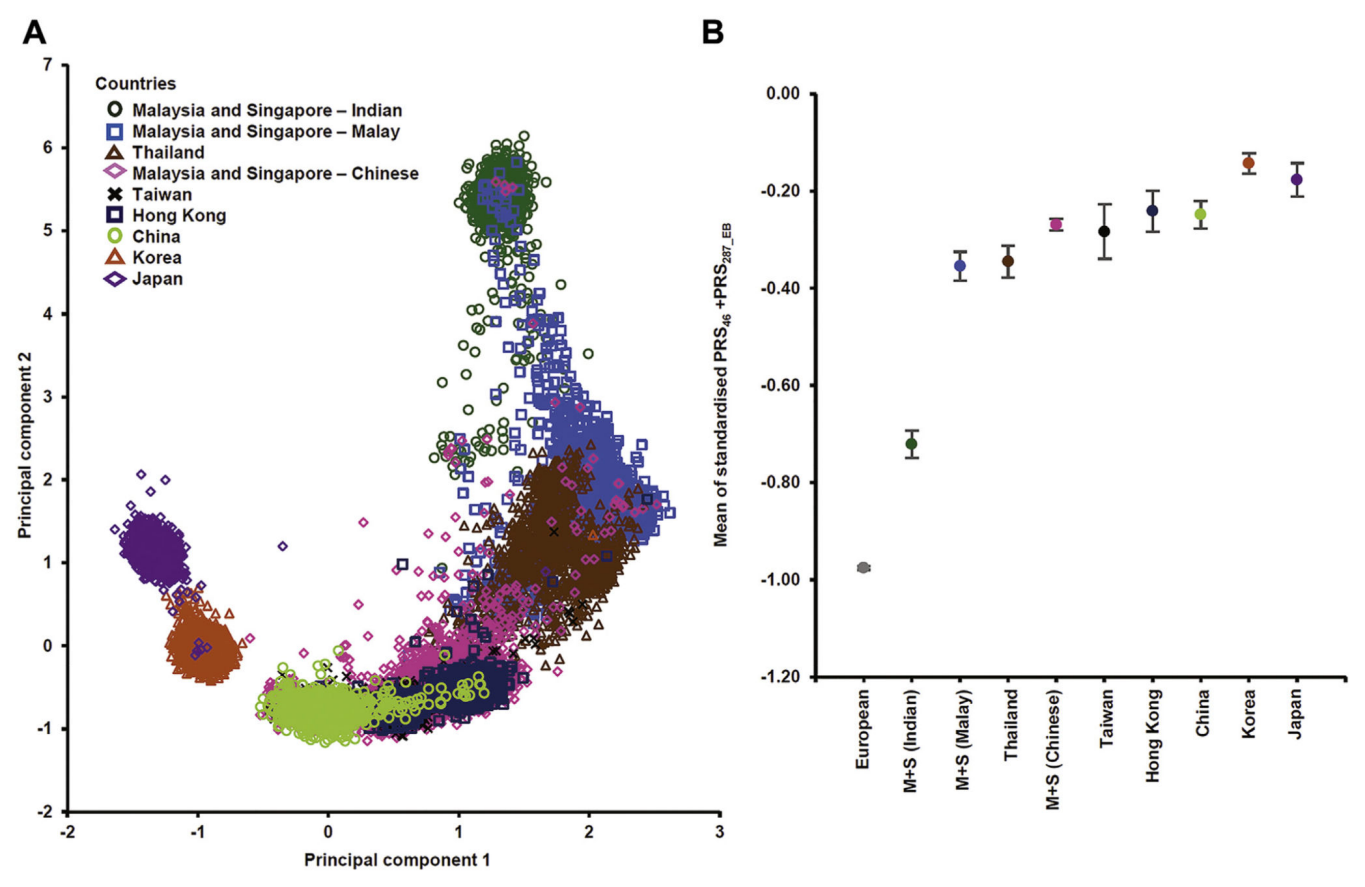
Principal components analysis and mean of PRS_46_ + PRS_287_EB_ according to country and ethnicity. (A) PC plotted according to country. PCs analysis of samples genotyped with OncoArray as listed in Supplemental Table 1. The samples were grouped according to country (Thailand, Taiwan, Hong Kong, China, Korea, and Japan). For M + S, the samples were further categorized by their self-reported ethnic origin (Chinese, Malay, and Indian). (B) Mean of standardized PRS_46_ + PRS_287_EB_ in controls according to country. PRS was standardized according to the control SDs of each study. Error bars represent 95% CI. The mean of standardized PRS_46_ + PRS_287_EB_ in European controls were included for reference. EB, Empirical Bayes; M + S, Malaysia and Singapore; PC, principal component; PRS, polygenic risk score.

Figure 1 summarizes the data set and methods used in this study. The populations are clustered, consistent with geography and population history, with the Chinese-ancestry women (Malaysia/Singapore/mainland China/Hong Kong/Taiwan) forming a distinct cluster that is genetically closer to Japanese/Koreans women than to Indian-ancestry women (Figure 2A). The Malay-ancestry women from Malaysia/Singapore are genetically closer to Chinese-ancestry women than to Indian-ancestry women. Given the large genetic distance between Indian-ancestry women from the other populations, the primary validation data set was based on Chinese-ancestry and Malay-ancestry women, and Indian-ancestry women were evaluated separately.

### PRSs developed using Asian-specific SNVs

For C + T, SNVs were removed if they were within 250 kb of an SNV already selected and correlated at *r* ^2^ > 0.1, leaving 1326 SNVs for analysis. For East Asian–ancestry women, the best PRS was obtained at a *P* value threshold of 5.74 × 10^−7^, resulting in a 46-SNV PRS (PRS_46_) (Supplemental Figure 2), with OR_perSD_ (95% CI) of 1.35 (1.30-1.39; AUC = 0.586) (Table 1). Other combinations of clumping size and correlation threshold *r* ^2^ did not result in PRSs that showed appreciable improvement (Supplemental Figure 3).

For lasso penalized regression, the best PRS was obtained at penalty parameter (λ) = 0.014 and shrinkage parameter (s) = 0.9, resulting in a PRS that included 2985 SNVs (PRS_2985_) (Supplemental Figure 4), with OR_perSD_ (95% CI) of 1.41 (1.36-1.46; AUC = 0.596), slightly more predictive than the PRS_46_ (Table 1).

### Linear combinations of European and Asian PRSs

Combining PRS287_EUR and PRS46 (OR_perSD_ [95% CI] = 1.54 [1.49-1.60]; AUC = 0.623) yielded markedly higher predictive accuracy in East Asian–ancestry women than that achieved using the Asian-specific PRSs alone (Table 1). The improvement was marginal when compared with the predictive accuracy obtained using PRS_287_EUR_ alone (OR_perSD_ [95% CI] = 1.50 [1.45-1.56]; AUC = 0.615), but relative contribution of PRS46 to the linear combination model was approximately 30% (Supplemental Table 5). Compared with PRS46 + PRS287_EUR, combining PRS287_EUR and PRS2985 further increased the OR_perSD_ and AUC.

### PRSs developed by integrating Asian weights into the European PRS

For East Asian-ancestry women, PRS_287_EB_ (OR_perSD_ [95% CI] = 1.53 [1.47–1.58]; AUC = 0.620) was slightly more predictive than PRS287_ASN (ORperSD [95% CI] = 1.50 [1.45-1.56]; AUC = 0.615) and PRS287_ EUR (ORperSD [95% CI] = 1.50 [1.45–1.56]; AUC = 0.614) and markedly more predictive than PRS_46_ and PRS_2985_ (Table 1). Compared with PRS46 + PRS287_EUR, a linear combination of PRS287_EB with PRS_46_ further improved the PRS performance.

### Continuous shrinkage PRSs (PRS-CSx)

The best combined PRS for East Asian–ancestry women was obtained at *φ* = 10^−4^ (Supplemental Table 6), with OR_perSD_ (95% CI) of 1.62 (1.52-1.68) and AUC of 0.636 for PRSGW_EUR + PRSGW_ASN, markedly better than all the PRSs described thus far (Table 1). This improvement was mainly driven by the contribution of PRS_GW_EUR_ (OR_perSD_ [95% CI] = 1.59 [1.53-1.65]; AUC = 0.629). The OR_perSD_ (95% CI) and AUC for PRSGW_ASN alone was 1.44 (1.39-1.49) and 0.601, respectively, only slightly better than PRS_46_ (Supplemental Table 6).

### PRSs for Indian-ancestry population

The PRSs derived from East Asian–ancestry women (as shown in Table 1) were all predictive of risk in South Asian–ancestry women but the OR_perSD_ were less than that of East Asian–ancestry women. Although linear combination of Asian-based and European-based PRSs improved the PRS performance compared with individual PRSs in East Asians, the improvement of PRS performance in women of South Asian ancestry was observed only when PRS_2985_ was considered in the linear combination (Table 2). There was no improvement in the effect sizes when European-based PRS was combined with PRS_46_. Whereas incorporating Asian weights via the EB approach improved the performance of PRSs in East Asians, there was no improvement in performance in women of South Asian ancestry. Re-estimating the weights of the combined models using South Asian–ancestry women in the validation data set did not lead to an appreciable difference in predictive performance (Supplemental Table 7).

### Evaluation of PRSs in prospective cohorts

The predictive performance of PRSs in the East Asian–ancestry women was replicated in the prospective cohorts (Table 1). Thus, the effect size was smallest for PRS based on Asian data alone (HR_perSD_ [95% CI] = 1.40 [1.25-1.56] for PRS_46_ and 1.45 [1.31-1.61] for PRS_2985_), larger for PRS based on the European PRS (HR_perSD_ [95% CI] = 1.50 [1.35-1.65] for PRS_287_EB_), and still larger for PRS based on combining the Asian and European PRS (HR_perSD_ [95% CI] = 1.53 [1.37-1.71] for PRS46 + PRS287_EB). As in the validation data set, PRS generated using PRS-CSx showed the strongest association with breast cancer risk (HR_perSD_ [95% CI] = 1.62 [1.46-1.80]) and highest AUC (0.635). There was no evidence of heterogeneity in the hazard ratios among studies for any PRS (Supplemental Figure 5).

### Absolute breast cancer risk predictions

**Figure 3:**
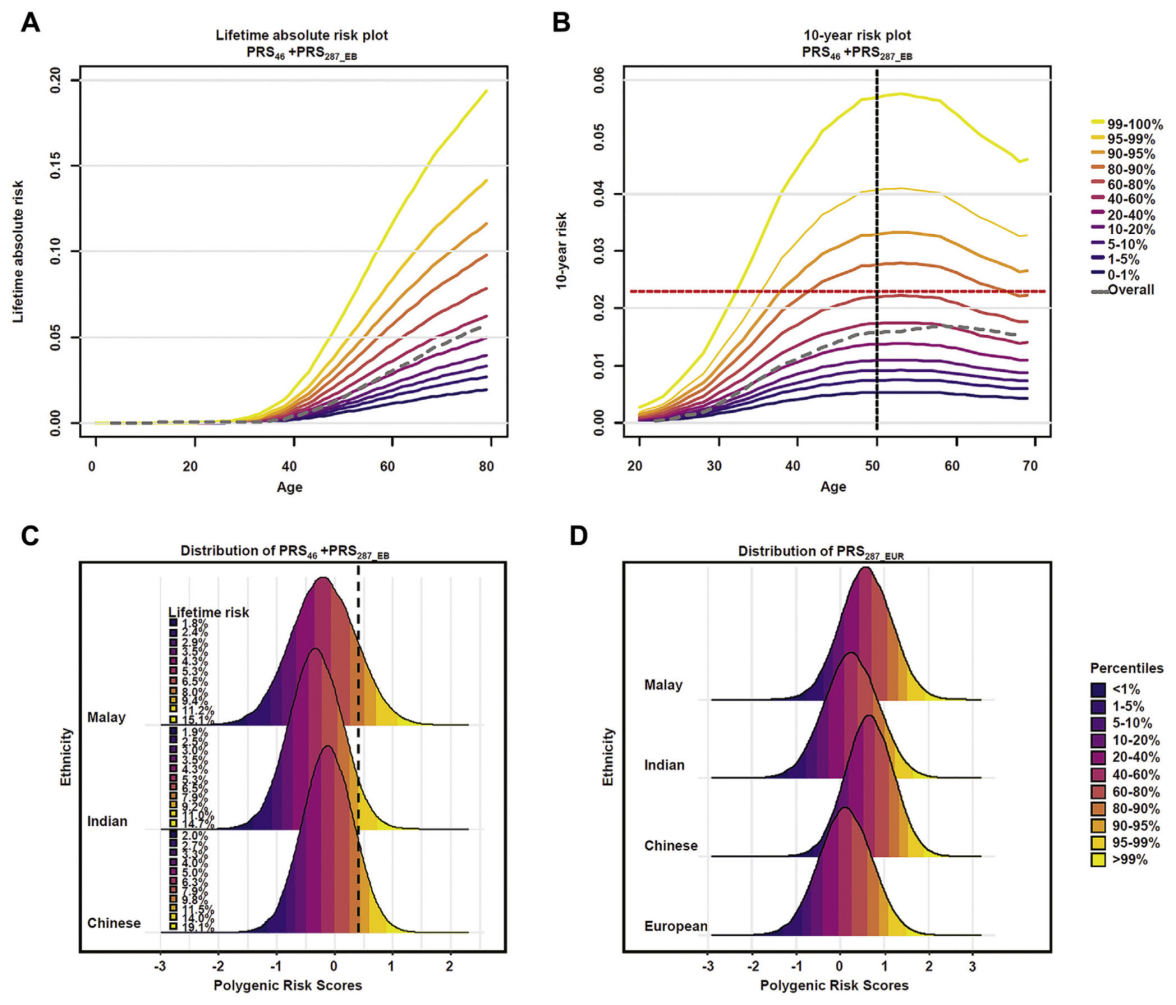
Absolute breast cancer risk by percentiles of PRS and PRS distribution by ancestry. (A) Lifetime and (B) 10-year absolute risk of developing breast cancer for Chinese women calculated using Singaporean incidence and mortality data and odds ratio per SD of PRS_46_ + PRS_287_EB_ in Chinese (1.56 as reported in Supplemental Table 9). The gray dashed lines in the (A) and (B) represent the average lifetime risk and absolute 10-year risk, respectively, for Singaporean Chinese women. The red horizontal dashed line (2.3%) in the (B) represents the 10-year absolute risk for a 50-year old EUR women where screening is recommended; (C) the distribution of PRS_46_ + PRS_287_EB_ in Chinese-ancestry, Indian-ancestry and Malay-ancestry women, generated using ethnic-specific mean and SD of controls as reported in Supplemental Table 9, and the corresponding cumulative breast cancer risk by age 80, generated using calendar-specific breast cancer incidence and mortality rates for Chinese, Malay, and Indian women in Singapore.^30^ Area under the curves represent the percentiles of PRS_287_EB_. The right vertical dashed line represents the 90th percentile cutoff for PRS distribution in Chinese-ancestry women; eg, the 95th percentile in Indians (lifetime risk = 11%) corresponds, approximately, to the 90th percentile in the Chinese population. If Chinese PRS distribution was used as a reference, these Indian women would be categorized as 90th percentile and hence would be told that their corresponding lifetime risk was 9% instead of 11%; (D) the distribution of EUR PRS (PRS_287_EUR_) for women of EUR ancestry, Chinese ancestry, Malay ancestry, or Indian ancestry. The right vertical dashed line represents the 90th percentile cutoff for PRS distribution in EUR-ancestry women. EB, Empirical Bayes; EUR, European; PRS, polygenic risk score.

We used PRS_46_ + PRS_287_EB_ to show the potential of translating PRS into clinical tool for Asian population. Based on East Asian-ancestry women in the validation data set, the estimated breast cancer ORs (95% CI) for women in the lowest 1% and highest 1% of the PRS distribution were 0.53 (0.33-0.82) and 3.01 (2.25-4.06), respectively, compared with middle quintile. The estimated ORs did not differ from those predicted under a theoretical polygenic model in which the log OR increases linearly with the PRS (Supplemental Table 8). The corresponding lifetime risks of developing breast cancer by age 80 years, on current incidence rates, were approximately 2% and approximately 19% for women in the lowest 1% and highest 1% of the PRS distribution, respectively, respectively (Figure 3A). Assuming that a 10-year absolute risk threshold of 2.3%^33^ is used to define women at sufficient risk to justify screening, approximately 12% of Chinese women would reach the risk threshold before or at age 40 (Figure 3B). Supplemental Figure 6 shows the distribution of the 10-year absolute risk at age 40 for women who were born between 1980 and 1989 using projected incidence rates (see Methods). It is projected that the proportion of women who would reach the risk threshold would rise to 29%.

### Generalizability of PRS across Asian ethnic subgroups

We showed the generalizability of PRS across Asian ancestry population using the 3 ethnic groups in the validation set and PRS_46_ + PRS_287_EB_ as an example. This combined PRS was predictive of risk in all ethnic groups, with the effect size being higher in Chinese-ancestry women than in Malay and Indian-ancestry women (OR_perSD_ [95% CI] = 1.56 [1.50-1.63] for Chinese vs 1.51 [1.39-1.64] for Malays and 1.49 [1.33-1.66] for Indians, heterogeneity *P* = .983) (Supplemental Figure 7, Supplemental Table 9). The PRS distribution was, however, different among the 3 ethnic groups. Although there was only a marginal difference in the SD, the means differed markedly, being highest in Chinese and lowest in Indians (mean [SD] in Chinese, Malay, and Indian controls were −0.118 [0.439], −0.197 [0.556], and −0.328 [0.455], respectively, P-values for pair-wise comparison of means < .0001) (Supplemental Table 9). Figure 3C shows that if the Chinese PRS distribution was applied to Indians without adjustment, the 95th percentile in Indians corresponds, approximately, to the 90th percentile in the Chinese population, resulting in underestimation of risk in Indian women. The difference in the PRS distributions is even more apparent when women of European ancestry is used as reference (Figure 3D).

The patterns of PRS distribution by population (Figure 2B) are mirrored in the genetic clusters shown in Figure 2A. The largest differences in the means of the standardized PRS_46_ + PRS_287_EB_ were observed between the Indian-ancestry women and Japanese/Korean women (with Indians being the biggest outlier).

## Discussion

Personalized risk stratification for prevention and early detection of breast cancer has gained increasing interest; however, it is important to recognize the need to study women representing diverse ancestries to lessen health disparities. Our study provides essential information about the utility of PRSs for breast cancer risk prediction in women of Asian ancestry. We developed and validated different PRSs for East Asian–ancestry women. The key observations were (1) PRSs generated by integrating information from European ancestry and Asian ancestry GWAS data sets performed better than PRSs based purely on weights derived from single-ancestry GWAS data, and (2) there were substantial differences in PRS distributions across ethnic groups.

Based on the largest available breast cancer GWAS data sets, the best PRS for East Asian–ancestry women was based on PRS-CSx approach^28^ (PRS_GW_EUR_ + PRS_GW_ASN_). This PRS had a notably larger effect size than the European PRS (PRS_287_EUR_) that we had previously shown to be the best breast cancer PRS for women of Asian ancestry^14^ (HR_perSD_ in prospective cohorts: 1.62 vs 1.46; Table 1). It is noteworthy that the predictive performance of this PRS was similar to that achieved in European populations (HR_perSD_ [95% CI] of 313-SNV PRS: 1.59 [1.54-1.64] as reported in Mavaddat et al^3^). However, despite the rapid drop in cost associated with next-generation sequencing, implementation of PRS comprising approximately 1 million SNVs can be practically more challenging than the implementation of the European PRS that included only 313 variants.

We showed that adaptions based on the European 313-SNV PRS can improve risk prediction in women of East Asian ancestry. First, incorporating SNVs identified in the Asian populations (PRS_46_) improved predictive power. This approach of linearly combining PRSs may reduce the gap in prediction accuracy between European and non-European populations as described previously.^34^ Second, incorporating Asian weights further improved predictive power (PRS_46_ + PRS_287_EB_) but to a lesser extent. The 313-SNV PRS is being used in several clinical studies in European populations, including the MyPeBs (My Personal Breast Screening)^8^ and WISDOM (Women Informed to Screen Depending On Measures of risk)^7^ trials, and the PRS_46_ + PRS_287_EB_ PRS would be relatively easy to implement in clinical settings.

The PRS generated for women of East Asian ancestry were also predictive for women of South Asian ancestry, but the effect sizes were smaller. When combining East Asian–derived genome-wide PRS with European-derived genome-wide PRS in women of South Asian ancestry using the PRS-CSx approach, it was noticeable that the East Asian component made a smaller contribution to the linear combination (relative contribution of approximately 14%, Supplemental Table 5). These results suggest the need for larger studies of women of South Asian ancestry both to optimize the PRS and validate in prospective cohorts.

One of the challenges of moving PRS into clinical implementation is transferability across different ethnic groups. Several studies have evaluated the population-level applicability of European PRSs to non-European populations for various diseases.^10,35–37^ Similar to these studies, we showed that the mean of the PRS distribution differ substantially between European and Asian ethnic subgroups. We showed that if the European PRS (PRS_287_EUR_) was applied to an Asian population without adjustment, the 60th percentile in Chinese-ancestry and Malay-ancestry women and 80th percentile in Indian-ancestry women correspond, approximately, to the 90th percentile in the European population, resulting in overestimation of risk in these women (Figure 3D). To our knowledge, no studies thus far have considered the transferability of breast cancer PRS within diverse Asian ethnic subgroups. Our results showed that although the effect sizes appeared to be similar across ethnic groups (Supplemental Table 8), the mean PRS distribution differed substantially across Asian populations (Supplemental Table 7, Figure 2B). For example, although Japanese, Koreans, and Han Chinese are conventionally classified as East Asians in genetic analyses, the mean PRSs were markedly different between these ethnic groups (Figure 2B). The differences are sufficiently large to affect risk classification, and thus, comparing the PRS for an individual woman with the correctly calibrated ethnic-specific distribution is crucial for valid risk prediction. This however can be problematic for admixed individuals, where the genomes composed from multiple ancestries that may be closely or distantly related to the reference population. As more samples of Asian ancestry become available, it may be possible to combine ethnic-specific PRSs with ancestry components to derive better multiethnic PRSs.^32^

Our work is subject to several limitations. First, although we have showed that the predictive performance of European PRS can be improved by integrating weights from Asians using an EB approach, the absolute increase in predictive accuracies is marginal. Second, our studies focus on developing PRS without using individual-level training data. When such data are available, it may be possible to develop PRS with higher accuracy using methods that fit all variants simultaneously, such as the step-wise hard-thresholding method as described in Mavaddat et al^3^ or considering subtype-specific disease analyses to retain more informative variants. Third, our results showed that PRSs developed using Asian-derived GWAS data set showed significantly poorer performance than the European PRS, indicating that further improvement is likely to require much larger Asian discovery data set. Finally, PRSs were linearly combined using the validation data set, and hence, the reported performance is likely subject to overfitting. Although we have shown that performance of the combined PRSs in East Asians were replicated in the prospective cohorts, we did not have a similar independent data set for South Asian women for such replication.

In summary, we have shown that genome-wide PRS derived from trans-ancestry method had significantly higher predictive accuracy for women of Asian ancestry than existing breast cancer PRSs. We also showed that European-based PRS can be improved for use in Asian populations by integrating population-specific weights and combined with Asian-specific PRS. Importantly, the differences in distribution of the same PRS across different ethnic groups (among Asians, and between Asian and Europeans) emphasize the need for ethnic-specific calibration before translating PRS into practice for diverse Asian populations.

## Supplementary Material

SuppMaterial

TableS1

TableS2

TableS3

TableS4

## Figures and Tables

**Table 1:** Mean, SD, and the association of PRSs with breast cancer risk in women of East Asian ancestry

Method	PRS	Validation Set^a^				Test Set^b^			
		Cases	Control	OR Per SD^c^		Cases	Control	HR Per SD^d^	
		Mean (SD)	Mean (SD)	(95% CI)	AUC	Mean (SD)	Mean (SD)	(95% CI)	AUC^d^
(1) Clumping and thresholding	^e^PRS_46_	−0.387 (0.446)	−0.538 (0.443)	1.37 (1.32-1.42)	0.589	−0.299 (0.433)	−0.444 (0.438)	1.40 (1.25-1.56)	0.600
(2) Penalized regression	^e^PRS_2985_	0.075 (0.455)	−0.082 (0.452)	1.41 (1.37-1.47)	0.598	0.107 (0.460)	−0.059 (0.458)	1.45 (1.31-1.61)	0.608
(3) EUR SNVs + EUR weights	^e^PRS_287_EUR_	0.865 (0.548)	0.640 (0.549)	1.50 (1.45-1.56)	0.615	0.876 (0.549)	0.679 (0.541)	1.46 (1.34-1.60)	0.609
(4) EUR SNVs + ASN weights	^e^PRS_287_ASN_	−0.533 (0.445)	−0.714 (0.447)	1.50 (1.45-1.56)	0.614	−0.552 (0.448)	−0.731 (0.441)	1.49 (1.33-1.66)	0.608
(5) EUR SNVs + EB weights	^e^PRS_287_EB_	0.343 (0.491)	0.135 (0.492)	1.53 (1.47-1.58)	0.620	0.341 (0.493)	0.153 (0.485)	1.50 (1.35-1.65)	0.609
Combine (1) + (3)	^f^PRS_46_ + PRS_287_EUR_	0.058 (0.440)	−0.134 (0.437)	1.54 (1.49-1.60)	0.623	0.103 (0.442)	−0.075 (0.436)	1.52 (1.36-1.70)	0.620
Combine (2) + (3)	^f^PRS_2985_ + PRS_287_EUR_	0.062 (0.447)	−0.139 (0.444)	1.56 (1.50-1.61)	0.626	0.080 (0.454)	−0.106 (0.447)	1.54 (1.38-1.72)	0.622
Combine (1) + (4)	^f^PRS_46_ + PRS_287_ASN_	0.052 (0.425)	−0.127 (0.423)	1.52 (1.47-1.58)	0.619	0.070 (0.425)	−0.113 (0.421)	1.52 (1.35-1.70)	0.621
Combine (2) + (4)	^f^PRS_2985_ + PRS_287_ASN_	0.055 (0.430)	−0.130 (0.430)	1.54 (1.48-1.60)	0.621	0.057 (0.435)	−0.135 (0.427)	1.53 (1.37-1.72)	0.623
Combine (1) + (5)	^f^PRS_46_ + PRS_287_EB_	0.061 (0.446)	−0.137 (0.443)	1.55 (1.50-1.61)	0.625	0.089 (0.447)	−0.089 (0.441)	1.53 (1.37-1.71)	0.621
Combine (2) + (5)	^f^PRS_2985_ + PRS_287_EB_	0.063 (0.451)	−0.139 (0.449)	1.56 (1.51-1.62)	0.627	0.077 (0.455)	−0.120 (0.447)	1.55 (1.39-1.72)	0.623
(6) PRS-CSx	^f^PRS_GW_EUR_ + PRS_GW_ASN_	0.082 (0.493)	−0.159 (0.489)	1.62 (1.52-1.68)	0.636	−0.145 (0.511)	−0.388 (0.511)	1.62 (1.46-1.80)	0.635

*ASN,* Asian; *AUC,* area under the receiver operating curve; *CKB,* China Kadoorie Biobank; *EB,* Empirical Bayes; *EUR,* European; *HR,* hazard ratio; *KCPS-II,* Korean Cancer Prevention Study-II Biobank; *MYBRCA,* Malaysian Breast Cancer Genetic Study; *OR,* odds ratio; *PRS,* polygenic risk score; *SCHS,* Singapore Chinese Health Study; *SGBCC,* Singapore Breast Cancer Cohort; *SNV,* single-nucleotide variation.

a Validation cohort that consisted of 6392 breast cancer cases and 6638 control of Chinese- and Malay-ancestry from MYBRCA and SGBCC (Supplemental Table 1).

b Prospective cohorts that consisted of 89,898 control and 1592 breast cancer cases from 3 prospective cohorts, SCHS, China Kadoorie Biobank (CKB), and KCPS-II (Supplemental Table 1).

c Adjusted for the first 10 principal components and study, and standardized to SDs in controls of each PRS.

d Fixed effect meta-analysis of 3 prospective cohorts, SCHS, CKB and KCPS-II. HR per SD and AUC of individual studies can be found in Supplemental Figure 5.

e PRSs were derived using 46, 2985 and 287 selected SNVs respectively as described in the Method section.

f Combined PRSs were generated using the formula *α*_0_ + *α*_1_ *PRS*_1_ + *α*_2_ *PRS*_2_ where *α*_0_, *α*_1_ and *α*_2_ are the weights obtained by fitting a logistic regression model with breast cancer as outcome, *PRS*_1_ and *PRS*_2_ as explanatory variables using the validation data set. The weights for the considered combination of PRSs can be found in Supplemental Table 5.

**Table 2:** Mean, SD, and the association of PRSs with breast cancer risk in women of South Asian ancestry

Method	PRS Developed on the basis of East Asians’data set^a^	Validation Set^b^			
		Cases	Control	OR Per SD^c^	
		Mean (SD)	Mean (SD)	(95% CI)	AUC
(1) Clumping and thresholding	^a^PRS_46_	−0.490 (0.388)	−0.548 (0.387)	1.18 (1.06-1.31)	0.546
(2) Penalized regression	^a^PRS_2985_	0.059 (0.381)	−0.048 (0.376)	1.32 (1.19-1.46)	0.581
(3) EUR SNVs + EUR weights	^a^PRS_287_EUR_	0.482 (0.570)	0.251 (0.608)	1.49 (1.34-1.67)	0.614
(4) EUR SNVs + ASN weights	^a^PRS_287_ASN_	−0.552 (0.493)	−0.720 (0.479)	1.43 (1.28-1.58)	0.592
(5) EUR SNVs + EB weights	^a^PRS_287_EB_	0.084 (0.521)	−0.127 (0.545)	1.50 (1.35-1.67)	0.613
Combine (1) + (3)	^d^PRS_46_ + PRS_287_EUR_	−0.212 (0.420)	−0.376 (0.444)	1.48 (1.33-1.65)	0.611
Combine (2) + (3)	^d^PRS_2985_ + PRS_287_EUR_	−0.166 (0.419)	−0.347 (0.441)	1.53 (1.37-1.71)	0.620
Combine (1) + (4)	^d^PRS_46_ + PRS_287_ASN_	0.008 (0.431)	−0.135 (0.420)	1.42 (1.28-1.57)	0.591
Combine (2) + (4)	^d^PRS_2985_ + PRS_287_ASN_	0.036 (0.425)	−0.121 (0.413)	1.46 (1.32-1.62)	0.602
Combine (1) + (5)	^d^PRS_46_ + PRS_287_EB_	−0.157 (0.438)	−0.328 (0.455)	1.49 (1.33-1.66)	0.610
Combine (2) + (5)	^d^PRS_2985_ + PRS_287_EB_	−0.119 (0.434)	−0.304 (0.449)	1.52 (1.37-1.70)	0.618
(6) PRS-CSx	^d^PRS_GW_EUR_ + PRS_GW_ASN_	−0.308 (0.501)	−0.546 (0.502)	1.62 (1.46-1.81)	0.633

*ASN,* Asian; *AUC,* area under the receiver operating curve; *EB,* Empirical Bayes; *EUR,* European; *MYBRCA,* Malaysian Breast Cancer Genetic Study; *OR,* odds ratio; *PRS,* polygenic risk score; *SGBCC,* Singapore Breast Cancer Cohort; *SNV,* single-nucleotide variation.

a PRSs developed on the basis of Chinese and Malay-ancestry women in the validation data set as described in Table 1. Cohort from Chinese- and Malay-ancestry of MYBRCA and SGBCC as in Table 1.

b Evaluation of PRSs performance in 585 breast cancer cases and 1018 controls of Indian-ancestry women in the validation dataset (Supplemental Table 1).

c Adjusted for the first 10 principal components and study, and standardized to SDs in controls of each PRS.

d Combined PRSs were generated using the formula *α*_0_ + *α*_1_ *PRS*_1_ + *α*_2_ *PRS*_2_ where *α*_0_, *α*_1_ and *α*_2_ are the weights estimated from East Asian ancestry women as described in Table 1. The weights for the considered combination of PRSs can be found in Supplemental Table 5.

## Data Availability

Summary statistics (odds ratios and confidence limits) for all single-nucleotide variations used in derivation of various polygenic risk scores are provided in Supplemental Tables 2 to 4 of the manuscript. Summary statistics of European breast cancer genome-wide association studies analysis used in this study can be accessed via Breast Cancer Association Consortium (BCAC) website (http://bcac.ccge.medschl.cam.ac.uk/bcacdata/oncoarray/oncoarray-and-combined-summary-result/). Summary statistics of genome-wide association studies analyses from The BioBank Japan Project can be accessed via The BioBank Japan Project website (https://pheweb.jp/pheno/BrC). Request for access to individual level data from BCAC studies can be made via the Data Access Coordinating Committee of BCAC (BCAC Coordinator: BCAC@medschl.cam.ac.uk). Request for access to the Asia Breast Cancer Consortium data could be requested by submission of an inquiry to Wei Zheng (wei.zheng@vanderbilt.edu).
