# Supplementary material for: Polygenic risk scores for prediction of breast cancer risk in Asian populations
Source: Genet Med. Author manuscript; Available in PMC 2022 Mar 8. (PMC7612481; doi:10.1016/j.gim.2021.11.008)
Supplement: SuppMaterial [file EMS140660-supplement-SuppMaterial.pdf]

Supplementary materials

Polygenic risk scores for prediction of breast cancer risk in Asian populations

Ho & Tai et al.

Figure S1. Schema for development and validation of PRS

The development of PRSs were conducted using clumping + thresholding method, lasso penalised regression, integration of Asian weights into European PRS, linear combinations of multiple PRSs and Bayesian polygenic prediction method. All the PRSs were subsequently evaluated in the prospective cohorts.

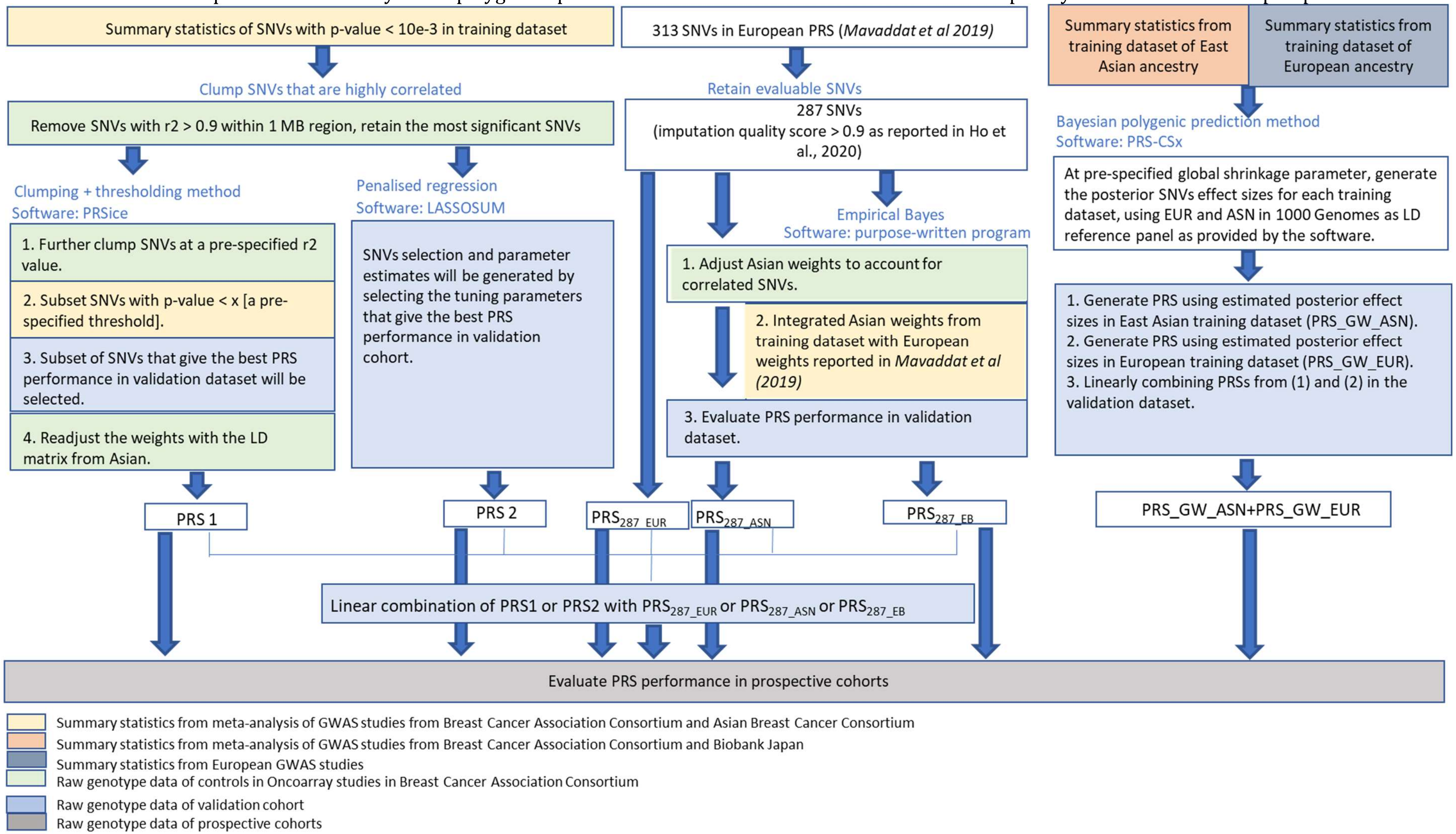

Figure S2. AUCs of PRSs generated using clumping and thresholding method for East Asian ancestry women

PRS performance (AUC) in validation dataset generated using clumping and thresholding method at different p-value threshold. Each point represents a p-value threshold. The best-fit PRS consisted of 46 SNVs at p-value threshold of 5.74 x 10^{-7}.

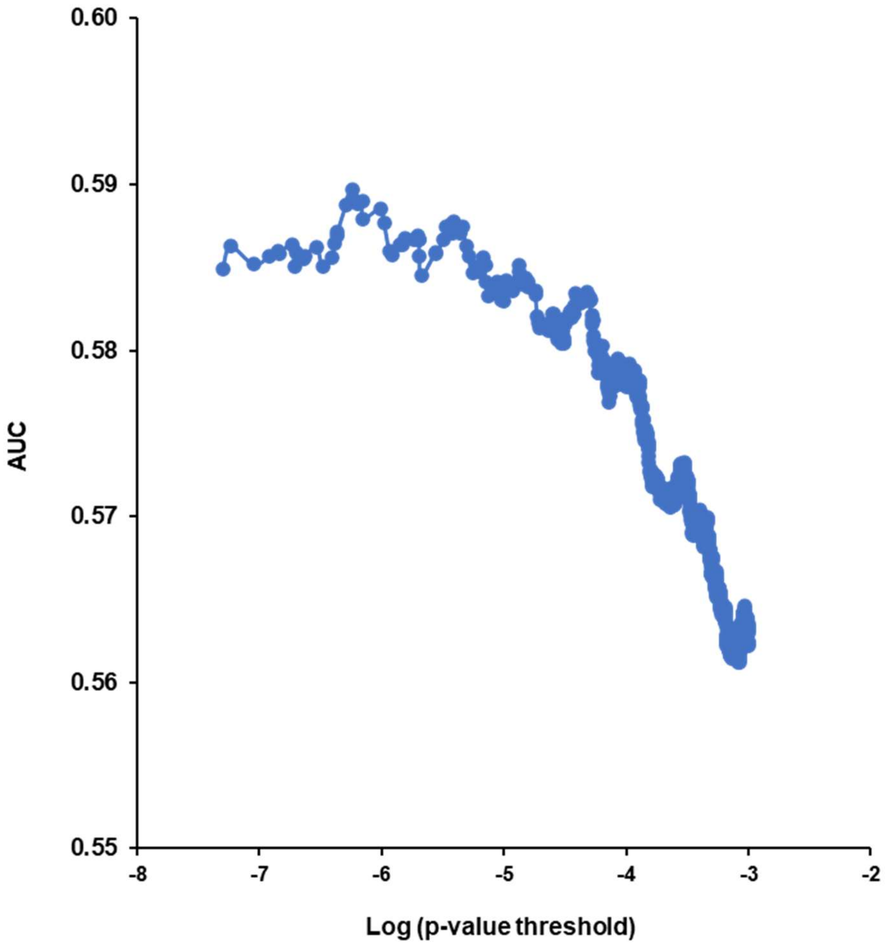

Figure S3. AUCs of best PRSs generated using clumping and thresholding method with different clumping option for East Asian ancestry women

Each point represents the AUC of the best PRS generate at the combination of the given clumping r^2 and clumping size.

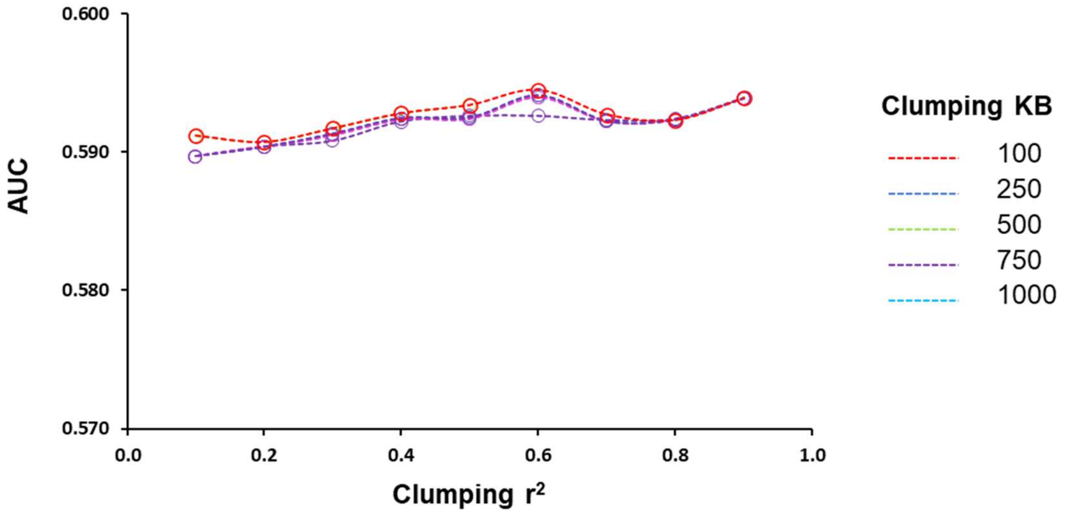

Figure S4. PRSs generating using different values of parameters in penalized regression for East Asian ancestry women

Each point represents correlation between breast cancer status in validation dataset and PRS generated at the given combination of penalty parameter (lambda) and shrinkage parameter (s). The best PRS occurred at penalty parameter (λ) equal to 0.014 and shrinkage parameter (s) equals to 0.9, where 2,985 SNVs were selected.

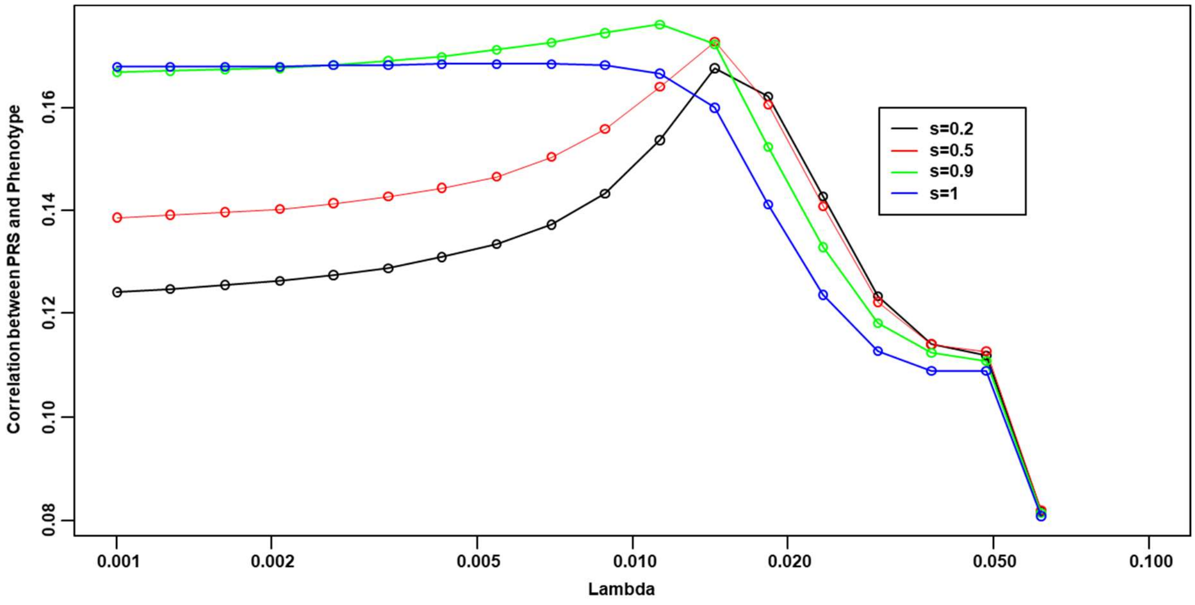

Figure S5. Performance of the PRSs in test dataset.

Forest plot showed the association between standardised PRSs and breast cancer risk in three prospective cohorts, China Kadoorie Biobank (CKB), Singapore Chinese Health Study (SCHS) and Korean Cancer Prevention Study II. The squares represent the hazard ratios (HRs), the horizontal lines represent the corresponding 95% confidence intervals and the diamond shapes represent the overall estimates. I-squared and p-value (two-sided) for heterogeneity were obtained by fitting a random-effects model and using generalized Q-statistic estimator (the rma() command in R).

(a) Performance of Asian-based and European-based PRSs

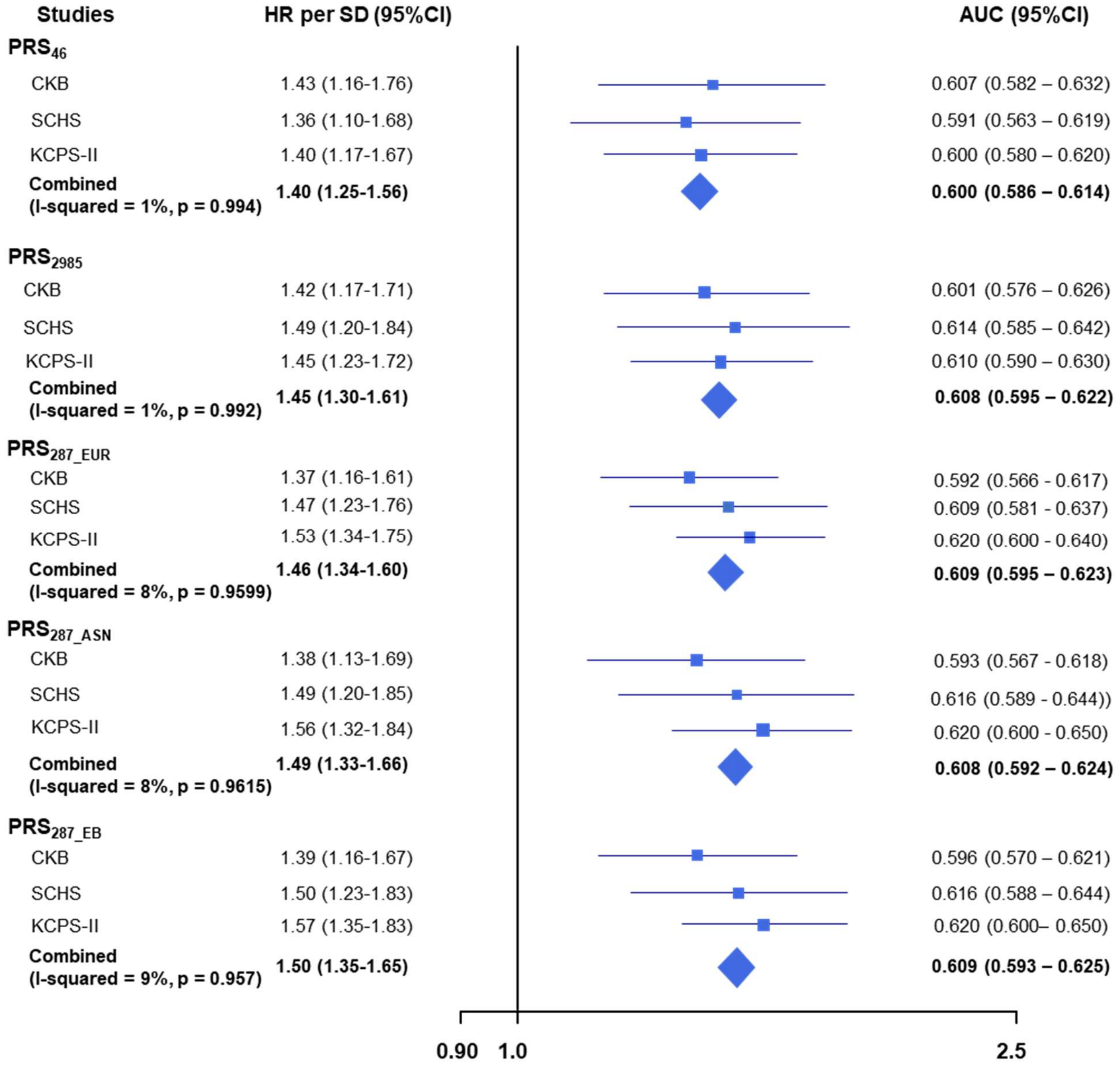

(b) Performance of PRSs generated by linear combinations of multiple PRSs.

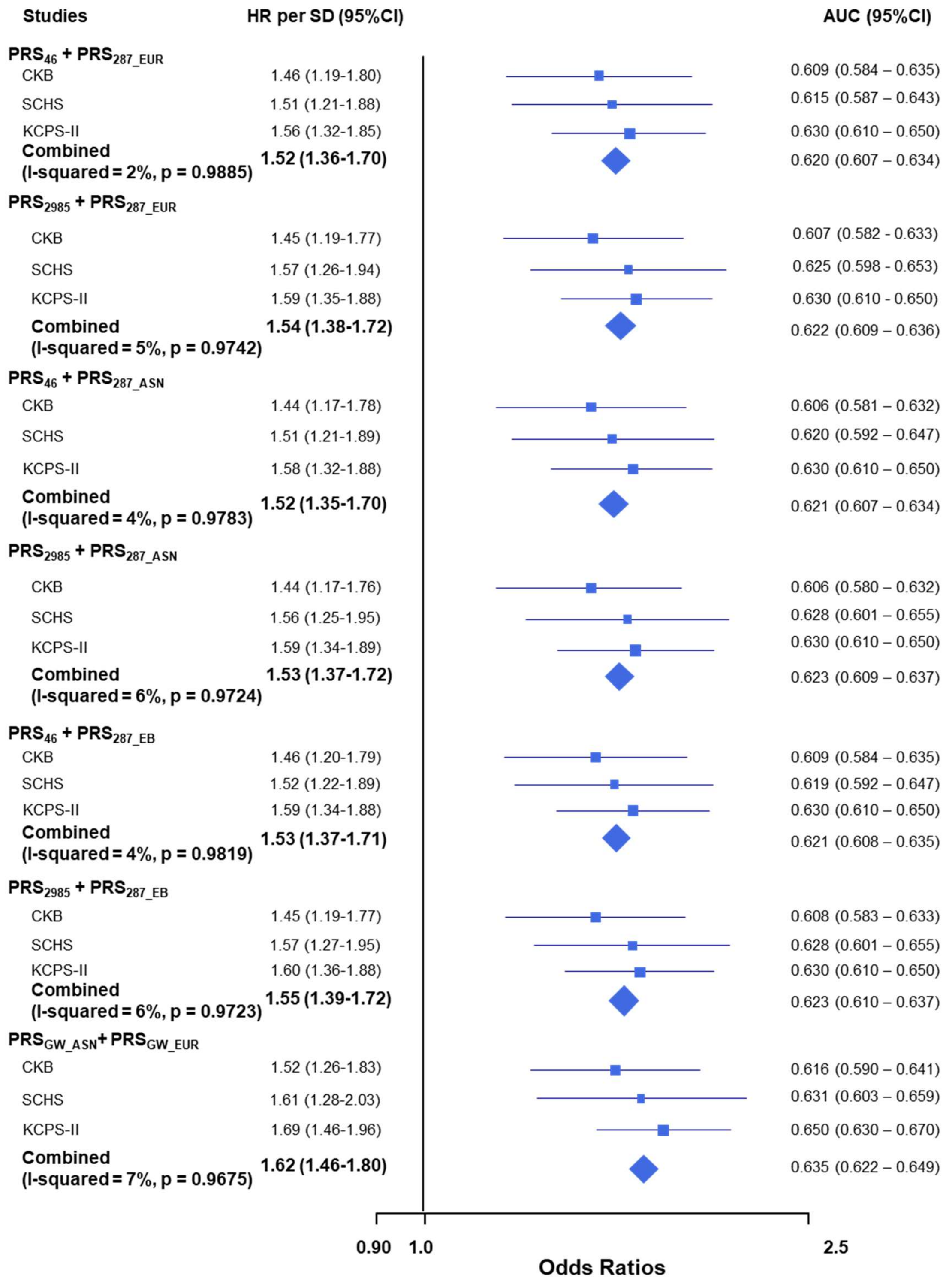

Figure S6. Distribution of 10-years absolute breast cancer risk at age 40 by birth cohort.

Dashed vertical line equals to 2.3% (average 10-year absolute risk of a 50-years old European women). Area under the curve represents the proportion of women who would have absolute risk at age 40 greater than a specific risk threshold. For example, area to the right of the vertical line for the blue curve represent proportion of women who were born after 1979 who would have absolute risk at age 40 greater than 2.3%. The breast cancer incidence for birth cohort 1960-1969 and 1970-1979 were observed and determined using breast cancer incidence in Singapore from 1968 to 2017. For birth cohort 1980-1989, breast cancer incidences were projected by assuming an annual increase in breast cancer incidence of 3.9%[1].

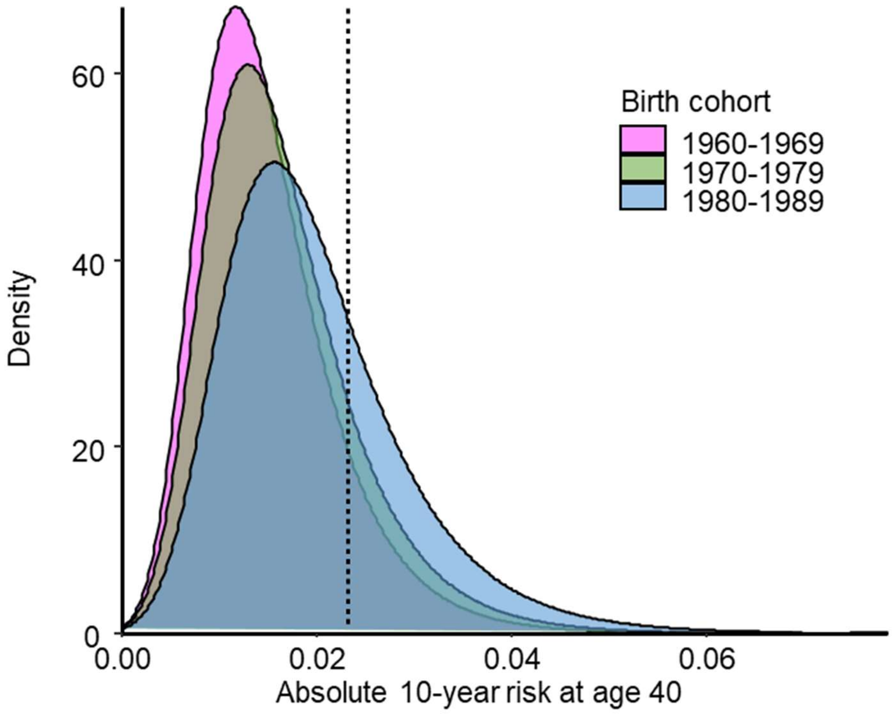

Figure S7. Performance of the PRS_{46} + PRS_{287_EB} in Chinese, Malay and Indian women from Malaysia and Singapore

Forest plot showed the association between standardised PRSs and breast cancer risk in Chinese, Malay and Indian women from Malaysia and Singapore (validation cohort). Odds ratios (ORs) and AUCs were generated using data from Malaysia Breast Cancer Genetics (MyBrCa) and Singapore Breast Cancer Cohort (SGBCC) studies, stratified by ethnicity. The squares represent the odds ratios (ORs), the horizontal lines represent the corresponding 95% confidence intervals and the diamond shapes represent the overall estimates. I-squared and p-value (two-sided) for heterogeneity were obtained by fitting a random-effects model and using generalized Q-statistic estimator (the rma() command in R). The number of cases and controls for each ethnicity, ORs and corresponding 95% confidence intervals are tabulated in Table S8.

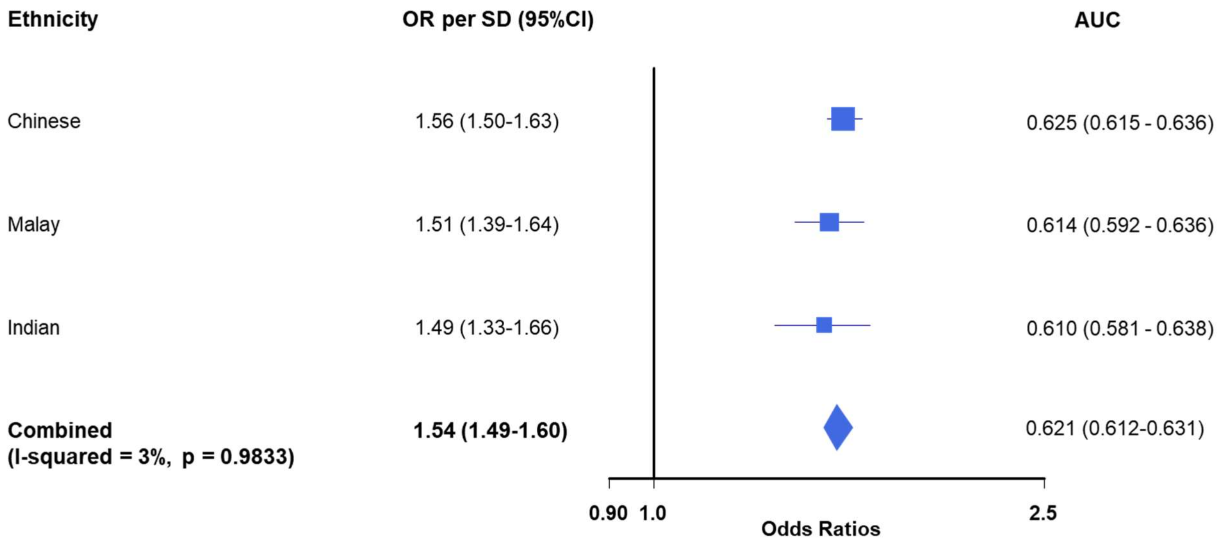

Please refer to attached excel for Table S1, S2, S3, and S4.

Table S1. Participating studies and the number of individuals used in polygenic risk scores evaluation analyses.

Table S2. SNVs and beta coefficient SNVs used in the construction of. PRS_{287}, PRS_{46}, PRS_{235} and PRS_{2985}.

Table S3. SNVs and beta coefficient SNVs used in the construction of. PRS_{GW_ASN}.

Table S4. SNVs and beta coefficient SNVs used in the construction of. PRS_{GW_EUR}.

Table S5. Weights used in the linear combinations of multiple PRSs

PRS combination	Weight, α_1 †	Weights, α_2 †	α_0 †	*w**
**East Asian ancestry**				
$\alpha_1 PRS_{46} + \alpha_2 PRS_{287\_EUR} + \alpha_0$	0.17389	0.33479	-0.31324	0.66
$\alpha_1 PRS_{2985} + \alpha_2 PRS_{287\_EUR} + \alpha_0$	0.19846	0.31648	-0.47000	0.61
$\alpha_1 PRS_{46} + \alpha_2 PRS_{287\_ASN} + \alpha_0$	0.14457	0.32984	0.57515	0.70
$\alpha_1 PRS_{2985} + \alpha_2 PRS_{287\_ASN} + \alpha_0$	0.17360	0.30808	0.39362	0.64
$\alpha_1 PRS_{46} + \alpha_2 PRS_{287\_EB} + \alpha_0$	0.14893	0.35354	-0.05224	0.70
$\alpha_1 PRS_{2985} + \alpha_2 PRS_{287\_EB} + \alpha_0$	0.17373	0.33482	-0.19889	0.66
$\alpha_1 PRS_{GW\_ASN} + \alpha_2 PRS_{GW\_EUR} + \alpha_0$	0.16856	0.38484	0.54881	0.70
**South Asian ancestry**				
$\alpha_1 PRS_{46} + \alpha_2 PRS_{287\_EUR} + \alpha_0$	0.03168	0.38924	-0.74574	0.92
$\alpha_1 PRS_{2985} + \alpha_2 PRS_{287\_EUR} + \alpha_0$	0.16089	0.33732	-0.75920	0.68
$\alpha_1 PRS_{46} + \alpha_2 PRS_{287\_ASN} + \alpha_0$	0.01452	0.33817	-0.08530	0.96
$\alpha_1 PRS_{2985} + \alpha_2 PRS_{287\_ASN} + \alpha_0$	0.16498	0.27290	-0.19377	0.62
$\alpha_1 PRS_{46} + \alpha_2 PRS_{287\_EB} + \alpha_0$	0.01339	0.39594	-0.52023	0.97
$\alpha_1 PRS_{2985} + \alpha_2 PRS_{287\_EB} + \alpha_0$	0.14950	0.33877	-0.54253	0.69
$\alpha_1 PRS_{GW\_ASN} + \alpha_2 PRS_{GW\_EUR} + \alpha_0$	0.07095	0.44277	0.39168	0.86

†Combined PRSs were generated using the formula $\alpha_0 + \alpha_1 PRS_1 + \alpha_2 PRS_2$ where α_0, α_1 and α_2 are the weights obtained by fitting a logistic regression model with breast cancer as outcome, and PRS_1 and PRS_2 are explanatory variables using East Asian ancestry women (top panel) or South Asian ancestry women (bottom panel) in the validation dataset. Here PRS_1 represents the Asian-based PRS and PRS_2 represent the European-based PRS. The PRSs were standardized to respective standard deviation (SD) of the controls in the validation dataset.

*Contribution of the European based PRS to the linear combination, where $w = \alpha_2 / (\alpha_1 + \alpha_2)$ and (1-w) represents the contribution of Asian based PRS to the linear combination.

Table S6. PRSs generated using different values of global shrinkage parameter in PRS-CSx for East Asian ancestry women

Global shrinkage parameter	PRS	East Asian Ancestries			
		Cases Mean (SD)	Control Mean (SD)	OR per SD† (95% CI)	AUC
ϕ=10^{-6}	PRS_{GW_ASN}	0.296 (0.109)	0.249 (0.108)	1.53 (1.48-1.59)	0.620
	PRS_{GW_EUR}	0.139 (0.169)	0.065 (0.169)	1.55 (1.50-1.61)	0.623
	*PRS_{GW_ASN} + PRS_{GW_EUR}	0.068 (0.460)	-0.143 (0.459)	1.58 (1.52-1.64)	0.628
ϕ=10^{-4}	PRS_{GW_ASN}	-0.142 (0.184)	-0.208 (0.183)	1.44 (1.39-1.49)	0.601
	PRS_{GW_EUR}	-0.183 (0.209)	-0.281 (0.211)	1.59 (1.53-1.65)	0.629
	*PRS_{GW_ASN} + PRS_{GW_EUR}	0.082 (0.492)	-0.158 (0.488)	1.62 (1.56-1.68)	0.636
ϕ=10^{-2}	PRS_{GW_ASN}	1.446 (0.513)	1.339 (0.516)	1.23 (1.19-1.28)	0.558
	PRS_{GW_EUR}	0.017 (0.432)	-0.135 (0.436)	1.41 (1.36-1.46)	0.597
	*PRS_{GW_ASN} + PRS_{GW_EUR}	0.346 (0.382)	-0.111 (0.382)	1.46 (1.41-1.51)	0.605
ϕ=1	PRS_{GW_ASN}	5.873 (0.995)	5.713 (1.005)	1.17 (1.13-1.21)	0.545
	PRS_{GW_EUR}	4.878 (0.848)	4.668 (0.846)	1.27 (1.22-1.31)	0.567
	*PRS_{GW_ASN} + PRS_{GW_EUR}	0.002 (0.283)	-0.078 (0.284)	1.31 (1.27-1.36)	0.579

†Adjusted for first the 10 principal components and study, and standardised to SDs in controls of each PRS.

*Combined PRSs were generated using the formula $\alpha_0 + \alpha_1 PRS_1 + \alpha_2 PRS_2$ where α_0, α_1 and α_2 are the weights obtained by fitting a logistic regression model with breast cancer as outcome, and PRS_1 and PRS_2 are explanatory variables using either East Asian ancestry women in the validation dataset. The PRSs were standardized to standard deviation (SD) of the controls in the validation dataset.

Table S7. Mean, standard deviation, and the association of polygenic risk scores (PRS) with breast cancer risk in women of South Asian ancestry

PRS developed based on South Asians	Validation cohort[a]			
	Cases Mean (SD)	Control Mean (SD)	OR per SD[†] (95% CI)	AUC
[b]PRS_{46} + PRS_{287_EUR}	-0.478 (0.376)	-0.631 (0.400)	1.50 (1.34-1.67)	0.614
[b]PRS_{2985} + PRS_{287_EUR}	-0.467 (0.404)	-0.641 (0.425)	1.53 (1.37-1.71)	0.620
[b]PRS_{46} + PRS_{287_ASN}	-0.493 (0.354)	-0.614 (0.344)	1.43 (1.28-1.59)	0.592
[b]PRS_{2985} + PRS_{287_ASN}	-0.482 (0.385)	-0.625 (0.374)	1.46 (1.32-1.63)	0.603
[b]PRS_{46} + PRS_{287_EB}	-0.477 (0.384)	-0.632 (0.401)	1.50 (1.35-1.67)	0.613
[b]PRS_{2985} + PRS_{287_EB}	-0.467 (0.409)	-0.641 (0.422)	1.53 (1.37-1.70)	0.618
[b]PRS_{GW_EUR} + PRS_{GW_ASN}	-0.438 (0.482)	-0.699 (0.480)	1.63 (1.47-1.83)	0.636

[a]Evaluation of PRSs performance in 585 breast cancer cases and 1,018 controls of Indian ancestry women in the validation dataset (Table S1).

[b]Combined PRSs were generated using the formula $\alpha_0 + \alpha_1 PRS_1 + \alpha_2 PRS_2$ where α_0, α_1 and α_2 are the weights obtained by fitting a logistic regression model with breast cancer as outcome, and PRS_1 and PRS_2 are explanatory variables using Indian-ancestry women in the validation dataset. The weights for the considered combinations of PRSs can be found in bottom panel of Table S5.

[†]Adjusted for first the 10 principal components and study, and standardised to SDs in controls of each PRS.

Table S8. Breast cancer odds ratio by percentiles of PRS_{46}+PRS_{287_EB} in East Asian ancestry women

Percentiles	Control	Cases	Estimated OR (95% CI)	Predicted OR
<1	67	29	0.53(0.33-0.82)	0.31
1-5	265	97	0.43(0.33-0.55)	0.35
5-10	232	137	0.49(0.39-0.60)	0.53
10-20	664	381	0.67(0.58-0.78)	0.63
20-40	1327	897	0.79(0.71-0.89)	0.79
40-60	1328	1134	1	1
60-80	1327	1505	1.33(1.19-1.48)	1.26
80-90	664	915	1.61(1.41-1.83)	1.58
90-95	232	544	1.91(1.63-2.24)	1.88
95-99	265	579	2.55(2.16-3.02)	1.85
>99	67	174	3.01(2.25-4.06)	3.24

PRS_{46}+PRS_{287_EB} was categorised into quantiles based on the PRS distribution in controls of validation dataset of East Asian ancestry women. The middle quintile was used as the reference category. Observed odds ratios (ORs) were compared with those predicted under a theoretical polygenic model in which the log OR depends-linearly on the PRS (see Supplementary Methods).

Table S9. Association between PRS_{46} + PRS_{287_EB} and breast cancer risk in validation cohort by ethnicity

Ethnicity*	Cases	Control	Cases Mean (SD)		Control Mean (SD)		OR per SD (95% CI)†	AUC (95% CI)
**PRS_{46}+PRS_{287_EB}**								
Chinese	5230	5153	0.076	(0.439)	-0.118	(0.439)	1.56 (1.50 - 1.63)	0.625 (0.615 - 0.636)
Malay	1086	1335	-0.007	(0.466)	-0.197	(0.556)	1.51 (1.39 - 1.64)	0.614 (0.592 - 0.636)
Indian	585	1018	-0.157	(0.438)	-0.328	(0.455)	1.49 (1.33 - 1.66)	0.610 (0.581 - 0.638)

†Adjusted for first 10 principal components and study, and standardised to SDs in controls of each PRSs.

*Self-declared ethnicity was used.

Supplementary Methods

Study populations and genotyping

The study population was divided into three datasets. PRSs were developed using training and validation datasets and evaluated in the testing dataset. The training dataset included women of East Asian ancestry from three sources: (a) 20,198 women (10,020 invasive cases and 10,179 controls) participating in 11 studies in Breast Cancer Association Consortium (BCAC); (b) 23,928 women (11,993 invasive cases and 11,935 controls) participating in 5 studies in the Asia Breast Cancer Consortium (ABCC); and (c) 79,550 (6,325 invasive cases and 73,225 controls) participating in Biobank Japan (BBJ). Except for studies in BCAC where raw genotype data was available, only summary statistics were available for the remaining studies. Training set 1 included summary statistics of variants with p-value < 10^{-3} from a meta-analysis of BCAC and ABCC studies as described in Shu et al, (2020)[30], training set 2 included summary statistics of variants from a meta-analyses of BCAC studies and BBJ. Given that summary statistics from ABCC studies were not available (only summary statistics of meta-analysed BCAC and ABCC studies were available) and a portion of samples in BBJ had been included in ABCC studies, we could not include ABCC studies in the meta-analysis of BCAC studies and BBJ. Finally, publicly available summary statistics from European GWAS were included as training set 3[23].

The validation set included 14,633 women of Easy Asian (Chinese-ancestry and Malay ancestry) or South Asian ancestry participating in two multi-ethnic case-control studies: (a) 6,993 women (3,384 invasive cases and 3,609 controls) participating in Malaysian Breast Cancer Genetics (MyBrCa) study; and (b) 7,640 women (3,593 invasive cases and 4,047

controls) participating in the Singapore Breast Cancer Cohort (SGBCC) study. Given that East Asians and South Asians are genetically distinguishable, we further divided the validation dataset into – set 1 included 13,030 (6,392 cases invasive cases and 6,638 controls) women of Chinese or Malay ancestry, and set 2 included 1,603 (585 invasive cases and 1,018 controls) women of Indian ancestry. Samples in the development dataset were genotyped using one of the five arrays: iCOGS[31], OncoArray[32], Affymetrix Genome-Wide Human SNV Array 6.0, Illumina Multi-Ethnic Genotyping Array, Illumina HumanOmiExpress[33] and Illumina HumanExome-12v1_A Beadchip[34-36] (Table S1).

The best PRSs were evaluated in the testing set comprising 89,898 women from three prospective cohorts of East Asian ancestry: (a) 10,021 women who had not had any cancer diagnosis prior to recruitment into Singapore Chinese Health Study (SCHS)[25,26] , of which 413 registry-confirmed breast cancer developed over 195,317 person years of prospective follow-up; (b) 38,864 women without any cancer diagnosis prior to recruitment into China Kadoorie Biobank (CKB)[28], of which 476 developed breast cancers over 423,396 person years of prospective follow-up; and (c) 41,031 without any cancer diagnosis prior to recruitment into Korean Cancer Prevention Study Biobank(KCPS-II)[29] of which 705 developed breast cancers over 406,556 person years of prospective follow-up. For all studies, follow-up started six months after recruitment and was censored at age of breast cancer diagnosis, age at risk-reducing mastectomy, age of diagnosis of any cancer, age of death, or age on 31 December 2015 (for SCHS), age on 31 December 2017 (for CKB) and age on 31 December 2017 (for KCPS-II) whichever came first. Samples in SCHS and KCPS-II were genotyped using the Illumina Global Screening Array[25,29], while samples in CKB were genotyped using custom-designed Affymetrix Axiom arrays[28]. Analyses using CKB data were conducted under research approval

2020-0047. Supplementary Table 1 summarises the study design and the number of breast cancer cases and controls in each study.

Genotype calling, quality control procedure and imputation have been described previously[23,30,31,33,37,38]. All data were imputed using the 1000 Genomes Project (Phase 3) data as the reference panel[39], except BioBank Japan, for which the HapMap Phase II (release 22)[40] was used. SNVs with overall minor allele frequency in controls > 0.01 and imputation $r^2 > 0.9$ for OncoArray studies, imputation $r^2 > 0.7$ for Biobank Japan and imputation $r^2 > 0.3$ for other studies in the training and validation dataset were included in this analysis. Since all samples in the validation sets were genotyped using OncoArray, a higher threshold was imposed for OncoArray to ensure accurate determination of PRS in the validation datasets.

Ancestry informative principal components were available for Asian ancestry samples in the training dataset and the validation dataset, generated using methods as previously described[30]. Briefly, for the BCAC data, continental ancestry was derived by combining the data with the 1000 Genomes Project reference data. Individuals with >40% estimated East Asian ancestry were retained. In the second stage, principal components were generated on the Asian ancestry individuals using a subset of uncorrelated SNVs. Similar ancestry informative principal components were generated for the other dataset.

All studies were approved by the relevant institutional ethics committees and review boards, and all participants provided written informed consent.

Single-SNV association analysis in the training set

Single-SNV association tests were conducted in the BCAC studies separately for the iCOGS and OncoArray datasets, adjusted for age, the first two principal components and country/study

to obtain the per-allele OR for each SNV using Plink 2.0[41]. Single-SNV association analyses in ABCC studies were previously conducted by Shu and colleagues (2020)[30] – only summary statistics from meta-analyses of BCAC and ABCC studies were available for this project. Briefly, the analyses were conducted separately for each study in ABCC, adjusted for age and first two principal components[30]. Combined weights and p-values were derived using fixed-effect meta-analysis with the software METAL[42]. A total of 20,768 SNVs significantly associated with breast cancer risk at p-values < 0.001 were selected. SNVs clumping (within 1Mb windows) was subsequently conducted using the software PRSice v2.11[43] to remove highly correlated SNVs (pairwise correlation $r^2 > 0.9$); the SNV with the lowest p-value for association in the correlated pairs was retained, resulting in 3,050 SNVs for subsequent analyses. Since the raw genotype data were not available for ABCC studies, the correlation r^2 was computed using 4,921 control samples in BCAC OncoArray studies only. Single-SNV association analyses in BBJ1 were previously conducted by *Ishigaki et al (2020)*[33]. Briefly, GWAS was conducted using generalised linear mixed model, adjusted for age and first five principal components. The GWAS summary statistics from BBJ were combined with GWAS from BCAC Asian studies using fixed-effect meta-analysis with the software METAL.

Clumping and thresholding (C+T) method

To account for the joint effect of SNVs used in derivation of the best PRS determined by the C+T method, the SNV weights should ideally be estimated jointly in a single logistic regression model. Raw genotype data of the training set were not available for the joint estimation. However, these weights can be computed from the marginal effect sizes – if $\underline{\gamma}$ are the (conditionally independent) effect sizes and $\gamma'_j = \gamma_j\sqrt{2p_j(1-p_j)}$ are the corresponding normalized effect sizes, where p_j is the effect allele frequency of SNV j, then the predicted

normalised marginal effect sizes of the SNVs β' are given by $\underline{\beta'} = R\underline{\gamma'}$, where R is the matrix of correlations between the SNV genotypes. Thus $\underline{\gamma'} = R^{-1}\underline{\beta'}$, and the optimal weight. The optimal weight for SNV *j* is then given by:

$$\gamma_j = {\gamma'}_j \Big/ \sqrt{2p_j(1-p_j)} \qquad (1)$$

The correlation matrix R was estimated using 4,921 Asian control samples in BCAC OncoArray studies. The concept of this method has been previously described in Section 3.3 of *Prive et al (2020)*[44].

Re-weighting of European-based PRS

For these analyses, we considered PRS based on the 313 SNVs developed in European women[45]. Of the 313 SNVs, only 287 SNVs with imputation info score > 0.9 in OncoArray studies were retained for subsequent analyses. We considered two sets of weights for these SNVs: (i) Asian weights estimated from training set 1 alone; and (ii) weights based on a combination of the Asian (from training set 1) and European weights, allowing for these weights to differ but be correlated.

For (i), the optimal weights taking into account the correlation between SNVs were derived using Equation (1). For (ii), we combined Asian and European weights using an Empirical Bayes approach. In brief, we assume that the true population-specific effect sizes vary from a "global" effect size, β_j, by a normally distributed amount, with variance ξ^2, i.e.

$$\beta'_{jA}, \beta'_{jE} \sim N(\beta_j, \xi^2)$$

where β'_{jA} and β'_{jE} are the unobserved true effect sizes for SNV *j* for Asian and European populations, respectively. Let $\gamma'_{jA} = \gamma_{jA}\sqrt{2p_j(1-p_j)}$ be the normalized weight of SNV *j*

estimated from the training set (using the method described above but p_j is the average effect allele frequency of SNV *j* in Asians and Europeans), and $\gamma'_{jE} = \gamma_{jE}\sqrt{2p_j(1-p_j)}$ be the normalized weight for SNV *j* reported for European populations (γ_{jE} are effect sizes reported in Mavaddat et al[45]). Using Bayes theorem and given that γ'_{jA} and γ'_{jE} are conditional independent given β'_{jA} the posterior distribution of $\beta'_{jA}|\gamma'_{jA}, \gamma'_{jE}$ is given by

$$f(\beta'_{jA}|\gamma'_{jA}, \gamma'_{jE}) \propto f(\gamma'_{jA}|\beta'_{jA}) f(\beta'_{jA}),$$

where $f(\gamma'_{jA}|\beta'_{jA}) \sim N(\beta'_{jA}, V_{\gamma'_{jA}})$ and $V_{\gamma'_{jA}}$ is the variance of γ'_{jA} estimated from the training dataset. Here, The estimated posterior effect sizes in Asians, given the data, are therefore:

$$\beta_{jA,EB} = \frac{\tau_A \gamma'_{jA} + \tau_\xi \beta_j}{\tau_A + \tau_\xi} \quad (2)$$

, $\tau_A = 1/V_{\gamma'_{jA}}$ and $\tau_\xi = 1/\xi^2$. The estimated posterior effect sizes for European populations, $\beta_{jE,EB}$, can be obtained using Equation (2) by replacing γ'_{jA} by γ'_{jE} and τ_A by τ_E, where $\gamma'_{jE} = \gamma_{jE}\sqrt{2p_j(1-p_j)}$ τ_E is the corresponding observed inverse variance of γ'_{jE}.

The parameters β_j, ξ^2 were estimated using Expectation-Maximisation (EM) iteration method where in the M-step, β_j was estimated using the formula

$$\beta_j = \frac{1}{2}(\beta_{jA,EB} + \beta_{jE,EB}),$$

since $\frac{1}{2}(\beta'_{jA} + \beta'_{jE})$ is an unbiased estimator of β_j, and the variance ξ^2 was derived using the following formula

$$\xi^2 = \frac{1}{2}\, var(\beta_{jA,EB} - \beta_{jE,EB}),$$

since $\beta'_{jA} - \beta'_{jE} \sim N(0, 2\xi^2)$. In the E-step, $\beta_{jA,EB}$ and $\beta_{jE,EB}$ were updated and the algorithm was repeated until β_j and ξ^2 converge. Finally, the optimal weights for each SNV included in PRS derivation were estimated using the following formula

$$\beta_{j,EB} = {\beta_{jA,EB}}\Big/{\sqrt{2p_j(1-p_j)}}.$$

This approach "shrinks" the Asian estimates towards the European estimates, making use of the greater precision in the European estimates but allowing for different Asian weights when the European and Asian estimates differ markedly.

Of the 287 SNVs, the combined Asian weights, $\hat{\beta}_{jA}$, in the training dataset was not available for 48 SNVs with MAF < 0.01, hence for these 48 SNVs, European weights, $\hat{\beta}_{jE}$, were used for PRS construction.

Absolute risk of breast cancer by PRS percentiles

The age-specific absolute risks of developing breast cancer in each PRS percentile, g, were calculated using the following formula:

$$\mathrm{AR_g(t)} = \sum_{\mathrm{u=0}}^{\mathrm{t}} \lambda_{\mathrm{g}}(\mathrm{u}) \cdot \mathrm{S_g(u)} \cdot \mathrm{S_m(u)}$$

where $\lambda_{\mathrm{g}}(u) = \lambda_0(u)\exp\,(\beta_g)$ is the breast cancer incidence associated with PRS at age u, $\lambda_0(u)$ is the baseline incidence and the corresponding effect size β_g, $S_g(u)$ is the probability of being breast cancer free at age u, and $S_m(u)$ is the probability of not dying from a cause other than breast cancer at age *u*. The theoretical effect sizes, $\mathrm{OR_g} = \exp(\beta_g)$, for PRS

interval between two percentiles (*u,v*), using middle quintile as reference (40-60th), were given by

$$\mathrm{OR_g} = \frac{(0.6-0.4)((\Phi(\Phi^{-1}(1-u)+\sigma)-\Phi(\Phi^{-1}(1-v)+\sigma))}{(\mathrm{v}-\mathrm{u})((\Phi(\Phi^{-1}(0.6)+\sigma)-\Phi(\Phi^{-1}(0.4)+\sigma))}$$

where σ is the log OR per unit SD of the continuous PRS[46]. The PRS-specific breast cancer incidences, $\lambda_g(u)$, were calculated iteratively by assuming that the average age-specific breast cancer incidence over all PRS percentiles agreed with the population breast cancer incidence. The details of these methods have been described previously[47]. We calculated lifetime and 10-year absolute risks using Singaporean mortality and breast cancer incidence for Chinese, Malays and Indians in 2017[48,49]. Under polygenic model, the logarithm of risk in the population has been shown to follow a normal distribution[50]. The proportion of population above a specific risk threshold was given by the area under the curve of the distribution of logarithm of 10-year absolute risk at pre-specified age.

To generate the distribution of birth-cohort specific 10-year absolute risk at age 40, we used birth cohort--specific breast cancer incidences derived using population breast cancer incidence in Singapore from 1968 to 2017[51]. The population incidences were reported in five-year age intervals for calendar year 1968-1972, 1973-1977,…, 2013-2017. By taking the lower-bound of these five-year age interval and the midpoint of calendar-specific interval, the year of birth of the cohort that the reported population incidences were based on were calculated. We took the average of the reported incidences according to three birth cohorts - 1960-1969, 1970-1979 and 1980-1989. Birth cohort-specific incidence were observed for women who were born between 1960-1969 and 1970-1979. For women who were born between 1980-1989, breast cancer incidence was observed up to age 35. The breast cancer incidence for this

birth cohort at age 40 was projected by assuming an annual increase in breast cancer incidence of 3.9%[1].

Supplementary References:

1. Jara-Lazaro, AR, Thilagaratnam S., and Tan PH, Breast cancer in Singapore: some perspectives. *Breast Cancer*, 2010. **17(1)**: p. 23-8.
2. Phuah SY, *et al.* Triple-negative breast cancer and PTEN (phosphatase and tensin homologue) loss are predictors of BRCA1 germline mutations in women with early-onset and familial breast cancer, but not in women with isolated late-onset breast cancer. *Breast cancer research : BCR* **14**, R142, doi:10.1186/bcr3347 (2012).
3. Wu AH, McKean-Cowdin R, Tseng CC. Birth weight and other prenatal factors and risk of breast cancer in Asian-Americans. *Breast Cancer Res Treat.* **14**:917–925 (2011).
4. Kawase T, *et al.* FGFR2 intronic polymorphisms interact with reproductive risk factors of breast cancer: results of a case control study in Japan. *International journal of cancer. Journal international du cancer* **125**, 1946-1952, doi:10.1002/ijc.24505 (2009).
5. Zheng W, *et al.* Genome-wide association study identifies a new breast cancer susceptibility locus at 6q25.1. *Nature genetics* **41**, 324-328, doi:10.1038/ng.318 (2009).
6. Lee KM, *et al.* Genetic polymorphisms of ataxia telangiectasia mutated and breast cancer risk. *Cancer Epidemiol Biomarkers Prev* **14**, 821-825, doi:10.1158/1055-9965.EPI-04-0330 (2005).
7. Hsu HM, *et al.* Breast cancer risk is associated with the genes encoding the DNA double-strand break repair Mre11/Rad50/Nbs1 complex. *Cancer Epidemiol Biomarkers Prev* **16**, 2024-2032, doi:10.1158/1055-9965.EPI-07-0116 (2007).
8. Ding SL, *et al.* Genetic variants of BLM interact with RAD51 to increase breast cancer susceptibility. *Carcinogenesis* **30**, 43-49, doi:10.1093/carcin/bgn233 (2009).
9. Grundy A, *et al.* Shift work, circadian gene variants and risk of breast cancer. *Cancer epidemiology* **37**, 606-612, doi:10.1016/j.canep.2013.04.006 (2013).
10. Kobayashi LC, *et al.* Moderate-to-vigorous intensity physical activity across the life course and risk of pre- and post-menopausal breast cancer. *Breast cancer research and treatment* **139**, 851-861, doi:10.1007/s10549-013-2596-9 (2013).
11. Grundy A, *et al.* Increased risk of breast cancer associated with long-term shift work in Canada. *Occupational and environmental medicine* **70**, 831-838, doi:10.1136/oemed-2013-101482 (2013).
12. Kobayashi LC, *et al.* A case-control study of lifetime light intensity physical activity and breast cancer risk. *Cancer causes & control : CCC* **25**, 133-140, doi:10.1007/s10552-013-0312-z (2014).
13. Kwong A, *et al.* Novel BRCA1 and BRCA2 genomic rearrangements in Southern Chinese breast/ovarian cancer patients. *Breast cancer research and treatment* **136**, 931-933, doi:10.1007/s10549-012-2292-1 (2012).
14. Kwong A, *et al.* Identification of BRCA1/2 founder mutations in Southern Chinese breast cancer patients using gene sequencing and high resolution DNA melting analysis. *PloS one* **7**, e43994, doi:10.1371/journal.pone.0043994 (2012).
15. Shimada N, *et al.* Genetic polymorphisms in estrogen metabolism and breast cancer risk in case-control studies in Japanese, Japanese Brazilians and non-Japanese Brazilians. *J Hum Genet* **54**, 209-215, doi:10.1038/jhg.2009.13 (2009).

16. John EM, *et al.* The Breast Cancer Family Registry: an infrastructure for cooperative multinational, interdisciplinary and translational studies of the genetic epidemiology of breast cancer. *Breast cancer research : BCR* **6**, R375-389, doi:10.1186/bcr801 (2004).
17. Han SA, *et al.* The Korean Hereditary Breast Cancer (KOHBRA) study: protocols and interim report. *Clin Oncol (R Coll Radiol)* **23**, 434-441, doi:10.1016/j.clon.2010.11.007 (2011).
18. Cho, Y.S. *et al.* A large-scale genome-wide association study of Asian populations uncovers genetic factors influencing eight quantitative traits. *Nat Genet* **41**, 527-34 (2009).
19. Han, S. *et al.* CASP8 polymorphisms, estrogen and progesterone receptor status, and breast cancer risk. *Breast Cancer Res Treat* **110**, 387-93 (2008).
20. Elgazzar, S. *et al.* A genome-wide association study identifies a genetic variant in the SIAH2 locus associated with hormonal receptor-positive breast cancer in Japanese. *J Hum Genet* **57**, 766-71 (2012).
21. Low, S.K. *et al.* Genome-wide association study of breast cancer in the Japanese population. *PLoS One* **8**, e76463 (2013).
22. Sakaue, S, & Kanai, M., et al. A global atlas of genetic assocaition of 220 deep phenotypes. *medRxiv* (2020)
23. Michailidou, K. et al. Association analysis identifies 65 new breast cancer risk loci. Nature 551, 92-94, doi:10.1038/nature24284 (2017)
24. Han S, *et al.* CASP8 polymorphisms, estrogen and progesterone receptor status, and breast cancer risk. *Breast cancer research and treatment* **110**, 387-393, doi:10.1007/s10549-007-9730-5 (2008).
25. Hankin JH, *et al.* Singapore Chinese Health Study: development, validation, and calibration of the quantitative food frequency questionnaire. Nutr Cancer: 39:187–195 (2001).
26. Wu AH, *et al.* Soy intake and breast cancer risk in Singapore Chinese Health Study. Br J Cancer: 99(1):196-200. doi: 10.1038/sj.bjc.6604448 (2008).
27. Pan, R. *et al.* Cancer incidence and mortality: A cohort study in China, 2008-2013. *Int J Cancer*.**1**;141(7):1315-1323 (2017).
28. Chen, Z. *et al.* China Kadoorie Biobank of 0.5 million people: survey methods, baseline characteristics and long-term follow-up. *Int J Epidemiol*.**40**(6):1652-66.(2011)
29. Jee, Y.H. *et al.* Cohort Profile: The Korean Cancer Prevention Study-II (KCPS-II) Biobank. *Int J Epidemiol.* **1**;47(2):385-386f (2018)
30. Shu, X. *et al.* Identification of novel breast cancer susceptibility loci in meta-analyses conducted among Asian and European descendants. *Nature communications* **11**, 1217, doi:10.1038/s41467-020-15046-w (2020).
31. Michailidou, K. *et al.* Large-scale genotyping identifies 41 new loci associated with breast cancer risk. *Nature genetics* **45**, 353-361, 361e351-352, doi:10.1038/ng.2563 (2013).
32. Amos, C. I. *et al.* The OncoArray Consortium: A Network for Understanding the Genetic Architecture of Common Cancers. *Cancer epidemiology, biomarkers & prevention : a publication of the American Association for Cancer Research, cosponsored by the American Society of Preventive Oncology* **26**, 126-135, doi:10.1158/1055-9965.EPI-16-0106 (2017).
33. Ishigaki, K. *et al.* Large-scale genome-wide association study in a Japanese population identifies novel susceptibility loci across different diseases. *Nat Genet*. 2020 Jul;**52(7)**:669-679.
34. Zhang, Y. *et al.* Rare coding variants and breast cancer risk: evaluation of susceptibility Loci identified in genome-wide association studies. *Cancer epidemiology, biomarkers & prevention : a publication of the American Association for Cancer Research, cosponsored by the American Society of Preventive Oncology* **23**, 622-628, doi:10.1158/1055-9965.EPI-13-1043 (2014).

35. Cai, Q. *et al.* Genome-wide association analysis in East Asians identifies breast cancer susceptibility loci at 1q32.1, 5q14.3 and 15q26.1. *Nature genetics* **46**, 886-890, doi:10.1038/ng.3041 (2014).
36. Zhang, B. *et al.* Large-scale genetic study in East Asians identifies six new loci associated with colorectal cancer risk. *Nature genetics* **46**, 533-542, doi:10.1038/ng.2985 (2014).
37. Dorajoo, R. *et al.* Loci for human leukocyte telomere length in the Singaporean Chinese population and trans-ethnic genetic studies. *Nature communications* **10**, 2491, doi:10.1038/s41467-019-10443-2 (2019).
38. Gan, W. *et al.* Evaluation of type 2 diabetes genetic risk variants in Chinese adults: findings from 93,000 individuals from the China Kadoorie Biobank. *Diabetologia* **59**, 1446-1457, doi:10.1007/s00125-016-3920-9 (2016).
39. Genomes Project, C. *et al.* A global reference for human genetic variation. *Nature* **526**, 68-74, doi:10.1038/nature15393 (2015).
40. International HapMap, C. *et al.* A second generation human haplotype map of over 3.1 million SNVs. *Nature* **449**, 851-861, doi:10.1038/nature06258 (2007).
41. Chang, C. C. *et al.* Second-generation PLINK: rising to the challenge of larger and richer datasets. *GigaScience* **4**, 7, doi:10.1186/s13742-015-0047-8 (2015).
42. Willer, C. J., Li, Y. & Abecasis, G. R. METAL: fast and efficient meta-analysis of genomewide association scans. *Bioinformatics* **26**, 2190-2191, doi:10.1093/bioinformatics/btq340 (2010).
43. Choi, SW, and O'Reilly PF. PRSice-2: Polygenic Risk Score software for biobank-scale data. *Gigascience*. 2019 Jul 1;**8(7)**:giz082.
44. Prive F, Arbel J and Vilhjalmsson BJ. LDpred2: better, faster, stronger. *Bioinformatics*2020 Dec 16;**36(22-23)**:5424-5431.
45. Mavaddat, N. *et al.* Polygenic Risk Scores for Prediction of Breast Cancer and Breast Cancer Subtypes. *American journal of human genetics* **104**, 21-34, doi:10.1016/j.ajhg.2018.11.002 (2019).
46. Wen, W. *et al.* Prediction of breast cancer risk based on common genetic variants in women of East Asian ancestry. *Breast Cancer Res* **18**, 124, doi:10.1186/s13058-016-0786-1 (2016).
47. Mavaddat, N. *et al.* Prediction of breast cancer risk based on profiling with common genetic variants. *JNCI: Journal of the National Cancer Institute* **107** (2015).
48. Cancer Incidence in Five Continents. (International Agency for Research on Cancer, Lyon, France, 2014).
49. *Age-Specific Death Rates, Annual* (Government of Singapore, 2017).
50. Pharoah, PDP *et al*. Polygenic susceptibility to breast cancer and implications for prevention. ***Nat. Genet***. **31**(1):33-6 (2002).
51. SINGAPORE CANCER REGISTRY 50TH ANNIVERSARY MONOGRAPH 1968 – 2017.
